# Supplementary material for: High-performing neural network models of visual cortex benefit from high latent dimensionality
Source: PLoS Comput Biol. 2024 Jan 10;20(1):e1011792. doi: 10.1371/journal.pcbi.1011792 (PMC10805290; doi:10.1371/journal.pcbi.1011792)
Supplement: S1 Text — Q&A-style response to questions that we anticipate readers to have. (PDF) [file pcbi.1011792.s001.pdf]

High-performing neural network models of visual cortex benefit from high latent dimensionality

Eric Elmoznino[*]
Department of Cognitive Science
Johns Hopkins University
Baltimore, MD 21218
eric.elmoznino@gmail.com

Michael F. Bonner
Department of Cognitive Science
Johns Hopkins University
Baltimore, MD 21218
mfbonner@jhu.edu

S1 - Anticipated questions

In this section, we address some questions that readers are likely to have regarding our results and conclusions. While certain parts of our responses are more speculative in nature and have yet to be tested empirically, we believe that they nevertheless provide useful insights.

Question: Isn't it trivially true that models with higher latent dimensionality will exhibit better encoding performance?

It is important to emphasize that the relationship between ED and encoding performance cannot be explained as a trivial statistical consequence of models with high ED. First, all models were evaluated using cross-validation, which means that the only way for a model to perform well is by explaining meaningful variance that generalizes to held-out data. If models with high ED were simply overfit to the training data, their performance on the held-out test data would be poor. Second, our ED metric characterizes the distribution of variance in the eigenspectrum, but it does not directly indicate the number of available dimensions in a system, nor does it change the number of parameters in the model. In fact, all models examined here were full rank, meaning that their image representations spanned the maximum number of latent dimensions. Thus, in our analyses, ED alone has no direct relationship to the maximum number of latent dimensions that could potentially be used in a regression. Finally, the data that we modeled come from a high-level visual region (IT) whose image-evoked responses have long been a challenging target for computational modelers. In fact, decades of efforts to model the representations in this brain region directly led to the advent of deep learning approaches for the computational neuroscience of vision [8, 3]. If any model with high ED could trivially explain the representations of IT, then neuroscientists would have no need for deep neural networks. One could, instead, solve the challenging problem of modeling IT by running linear regression on RGB pixel values and adding polynomials or interaction terms until ED was high enough to account for the variance in neural responses. The reason that such an approach would not work is that the space of all possible image representations is infinite: there is an unlimited variety of arbitrary computations that could be used to add dimensions to a model. Models that achieve high ED through arbitrary computations would have a negligible probability of overlapping with the representations of visual cortex. We, thus, suspect that the use of performance-optimized DNN architectures is critical for constraining the computations of encoding models and increasing their overlap with cortical representations.

Question: Why should we care about latent dimensionality if ImageNet classification performance also correlates with encoding performance?

Prior work has found that a model's object classification accuracy (e.g., on ImageNet) strongly correlates with its encoding performance for certain brain regions [8, 9]. Could we simply focus on classification accuracy, or is latent dimensionality theoretically important in its own right?

[*]Corresponding author.

While object classification accuracy seems to account for the encoding performance of current models, it is worth asking whether or not it can be a viable theory going forward. To say that it is a complete explanation is to say that we believe the correlation between classification accuracy and encoding performance will hold indefinitely, across the space of all current and future models (i.e., a model that performs better on object classification will always obtain higher encoding performance). This is unlikely to be the case. Optimal object classification requires that a model be invariant to features unrelated to object identity, such as orientation and position, which can only contribute to noise in the classifier [4]. However, we know that the brain represents orientation, position, and a host of other features unrelated to object identity. Therefore, we know that the object classification theory of encoding performance breaks down in some regime, and that the true dimensionality of visual cortex must be higher than what ideal object classification models would predict. Indeed, initial results suggest that the relationship between object classification and encoding performance does indeed break down past a certain ImageNet classification accuracy [5, 6]. A theory based on latent dimensionality (and alignment pressure) has the potential to explain the encoding performance of both current and future models on more rich neural datasets, and it may help us to understand why the relationship between encoding performance and classification accuracy breaks down at the highest levels of classification performance.

An interesting question emanating from this discussion is whether the observed relationship between classification accuracy and encoding performance might be overly optimistic due to the limited space of DNNs that are available for computational neuroscientists to examine. Most of the DNNs in visual neuroscience are trained on ImageNet or similar image databases, and we do not have DNNs that can perform open-ended tasks in complex, real-world environments. If we did have DNNs that handled more complex and naturalistic visual behaviors, we postulate that they would surpass the encoding performance of our best object-classification models (and also have higher dimensionality). With the current space of state-of-the-art DNNs being dominated by (a) supervised object classification and (b) self-supervised objectives that learn invariances tailored to object classification, we are bound to observe the current correlation between object classification performance and encoding performance because object recognition is undoubtedly *one* important problem that biological vision solves—but, importantly, it is one of *many* complex problems solved by the representations of visual cortex.

Finally, ED and classification performance are two fundamentally different *levels* of explanation that are mutually compatible because high classification accuracy itself is explained by certain geometric properties of a representation, of which ED is an important one (see Sections 2.4 and 2.5 in the main manuscript). We elaborate on this point in S10 where we examine the relationship between classification performance, encoding performance, and ED in our models.

Question: Does effective dimensionality really represent the number of accurately encoded visual features?

Essential to our theory is the assumption that the variance of a representation along a particular dimension is proportional to the meaningfulness of the feature it encodes. In such cases, it is valid to say that ED roughly quantifies the number of encoded visual features. This interpretation is central to popular dimensionality reduction techniques, such as PCA, and it has good theoretical support given that high-variance dimensions are typically more robust and would, thus, be best-suited for carrying the signal in a population code. Importantly, in the DNN literature, recent findings have shown that neural networks expand variance along dimensions that are useful for solving their tasks and contract variance along noise dimensions that are left over from their random initialization [2, 4, 1]. There is, thus, a straightforward relationship between the number of meaningful latent dimensions in a neural network and the shape of the principal component variance spectrum.

Nonetheless, there is no guarantee that high-variance dimensions correspond to meaningful signal and that low-variance dimensions correspond to random features or noise. Furthermore, ED is only an imperfect summary statistic of the rate of decay of the eigenspectrum, and does not directly quantify the number of meaningful features. An interesting direction for future work would be the development of dimensionality metrics that try to explicitly differentiate between meaningful and random dimensions in DNNs, as can be done for neural data when using repeated stimulus presentations [7].

References

[1] Timo Flesch, Keno Juechems, Tsvetomira Dumbalska, Andrew Saxe, and Christopher Summerfield. Orthogonal representations for robust context-dependent task performance in brains and neural networks. *Neuron*, 110(7):1258–1270.e11, 2022. ISSN 0896-6273. doi: https://doi.org/10.1016/j.neuron.2022.01.005. URL https://www.sciencedirect.com/science/article/pii/S0896627322000058.

[2] Spencer Frei, Niladri S. Chatterji, and Peter L. Bartlett. Random feature amplification: Feature learning and generalization in neural networks, 2022. URL https://arxiv.org/abs/2202.07626.

[3] N. Kriegeskorte. Deep Neural Networks: A New Framework for Modeling Biological Vision and Brain Information Processing. *Annu Rev Vis Sci*, 1:417–446, Nov 2015.

[4] Stefano Recanatesi, Matthew Farrell, Madhu Advani, Timothy Moore, Guillaume Lajoie, and Eric Shea-Brown. Dimensionality compression and expansion in deep neural networks. *CoRR*, abs/1906.00443, 2019. URL http://arxiv.org/abs/1906.00443.

[5] Martin Schrimpf, Jonas Kubilius, Ha Hong, Najib J. Majaj, Rishi Rajalingham, Elias B. Issa, Kohitij Kar, Pouya Bashivan, Jonathan Prescott-Roy, Franziska Geiger, Kailyn Schmidt, Daniel L. K. Yamins, and James J. DiCarlo. Brain-score: Which artificial neural network for object recognition is most brain-like? *bioRxiv preprint*, 2018. URL https://www.biorxiv.org/content/10.1101/407007v2.

[6] Martin Schrimpf, Jonas Kubilius, Michael J Lee, N Apurva Ratan Murty, Robert Ajemian, and James J DiCarlo. Integrative benchmarking to advance neurally mechanistic models of human intelligence. *Neuron*, 2020. URL https://www.cell.com/neuron/fulltext/S0896-6273(20)30605-X.

[7] Carsen Stringer, Marius Pachitariu, Nicholas Steinmetz, Matteo Carandini, and Kenneth D. Harris. High-dimensional geometry of population responses in visual cortex. *Nature*, 571(7765):361–365, Jul 2019. ISSN 1476-4687. doi: 10.1038/s41586-019-1346-5. URL https://doi.org/10.1038/s41586-019-1346-5.

[8] Daniel L. K. Yamins, Ha Hong, Charles F. Cadieu, Ethan A. Solomon, Darren Seibert, and James J. DiCarlo. Performance-optimized hierarchical models predict neural responses in higher visual cortex. *Proceedings of the National Academy of Sciences*, 111(23):8619–8624, 2014. ISSN 0027-8424. doi: 10.1073/pnas.1403112111. URL https://www.pnas.org/content/111/23/8619.

[9] Chengxu Zhuang, Siming Yan, Aran Nayebi, Martin Schrimpf, Michael C. Frank, James J. DiCarlo, and Daniel L. K. Yamins. Unsupervised neural network models of the ventral visual stream. *Proceedings of the National Academy of Sciences*, 118(3), 2021. ISSN 0027-8424. doi: 10.1073/pnas.2014196118. URL https://www.pnas.org/content/118/3/e2014196118.
