# Supplementary material for: High-performing neural network models of visual cortex benefit from high latent dimensionality
Source: PLoS Comput Biol. 2024 Jan 10;20(1):e1011792. doi: 10.1371/journal.pcbi.1011792 (PMC10805290; doi:10.1371/journal.pcbi.1011792)
Supplement: S2 Text — Detailed description of the theory of ED and encoding performance sketched out in Section Dimensionality and alignment in computational brain models. (PDF) [file pcbi.1011792.s002.pdf]

High-performing neural network models of visual cortex benefit from high latent dimensionality

Eric Elmoznino*
Department of Cognitive Science
Johns Hopkins University
Baltimore, MD 21218
eric.elmoznino@gmail.com

Michael F. Bonner
Department of Cognitive Science
Johns Hopkins University
Baltimore, MD 21218
mfbonner@jhu.edu

S2 - Theory of latent dimensionality and encoding performance

While the space of all possible visual stimuli is vast and high-dimensional, we can define many lower-dimensional subspaces within it, referred to as *subspaces* (Fig S2.1a). For instance, the *natural image subspace* consists solely of images taken from the physical world, and typical neuroscience experiments consist of tightly controlled stimulus sets spanning a *data subspace*. In a similar way, we can formalize visual representations and the features they encode using the framework of subspaces embedded in a higher-dimensional visual space.

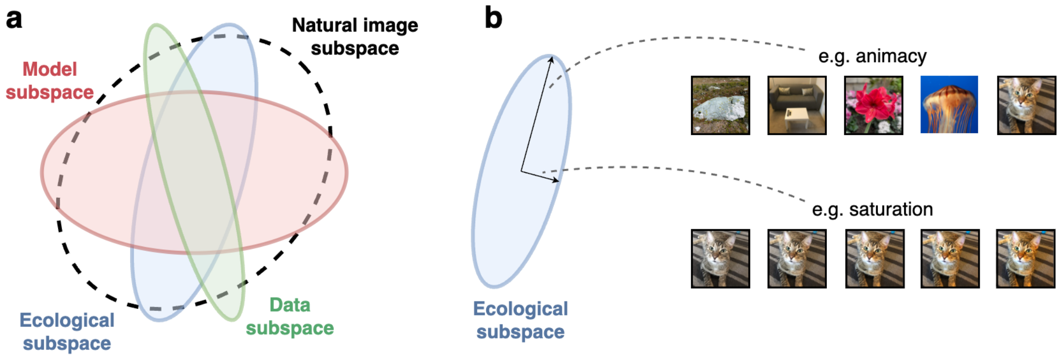

Supplementary Figure S2.1: A theory of latent dimensionality and encoding performance. a. Our theory models the distribution of natural images, the distribution of experimental stimuli, and the features encoded by brains and models as lower-dimensional subspaces embedded in a high-dimensional ambient space (denoted here using ellipses of varying eccentricity). **b.** For ecological and model subspaces, the variance along a dimension represents the accuracy with which it is encoded. For example, visual cortex might accurately encode differences in animacy (high variance), but only coarsely encode differences in color saturation (low variance).

For instance, if we were to show a human subject a set of object images that varied along the dimension of animacy (e.g., ranging from inanimate rocks to cats) we would expect them to clearly and accurately notice the differences between these images (see Fig S2.1b). On the other hand, if we were to vary a more perceptually subtle property, such as color saturation, the differences might appear less pronounced and we could say that the visual system is less accurate along this dimension. At the extreme, we could generate images by sampling from a distribution of white noise, in which case differences would be almost imperceptible, despite varying significantly in the input space.

What these examples show is that the human brain does not accurately encode the vast space of all possible visual dimensions, but only a lower-dimensional subspace of these dimensions that

*Corresponding author.

are ecologically relevant for survival and behavior in the real world (i.e., dimensions along an *ecological subspace*). In the same way, any computational model of perception, such as a DNN, will preferentially encode different visual dimensions more or less accurately and define its own representational *model subspace*. We refer to dimensions along these representational subspaces as *latent*, and we refer to the dimensions of the larger visual space in which they are embedded as *ambient*.

With these concepts in mind, we can now begin to think about the conditions under which a model can achieve high encoding performance when predicting a given neural dataset. Intuitively, this can only happen when the stimuli span dimensions that are accurately encoded by *both* the model and the brain. In other words, the latent dimensions of the ecological subspace must overlap with latent dimensions of the model subspace. A key factor driving our simulated and empirical results is that the probability of these overlaps increases substantially as the latent dimensionality of the model subspace grows.
