# Supplementary material for: High-performing neural network models of visual cortex benefit from high latent dimensionality
Source: PLoS Comput Biol. 2024 Jan 10;20(1):e1011792. doi: 10.1371/journal.pcbi.1011792 (PMC10805290; doi:10.1371/journal.pcbi.1011792)
Supplement: S3 Text — Details for how we generated the simulated data and fit encoding models in Section Dimensionality and alignment in computational brain models. (PDF) [file pcbi.1011792.s003.pdf]

High-performing neural network models of visual cortex benefit from high latent dimensionality

Eric Elmoznino*
Department of Cognitive Science
Johns Hopkins University
Baltimore, MD 21218
eric.elmoznino@gmail.com

Michael F. Bonner
Department of Cognitive Science
Johns Hopkins University
Baltimore, MD 21218
mfbonner@jhu.edu

S3 - Implementation of simulations

Our process is summarized in Fig S3.1, and consists of 3 steps. First, given our simulation parameters, we sampled subspace geometries within a high-dimensional ambient space. Next, we generated a set of experimental stimuli from the data subspace and projected them onto both the ecological and model subspaces, yielding a set of neural and model activations. Finally, we fit a linear encoding model to measure how well neural responses to the stimuli could be predicted from the corresponding model activations. By repeating this process across a range of simulation parameters (e.g., different model effective dimensionalities), we could better understand their influences on encoding performance. We describe this process in more detail below.

Subspace geometry In essence, our simulations consider four subspaces and relations between them: the natural image subspace, the data subspace from which experimental stimuli are sampled, the ecological subspace governing neural representations, and the model subspace. For simplicity, we parameterized all subspaces as multivariate Gaussian distributions embedded in a common ambient space of all possible visual dimensions. Using multivariate Gaussians also provided a simple method for modulating and measuring the subspace's latent dimensionality, which we describe later in this section.

Variance along latent dimensions In our simulations, the amount of variance in a subspace along a given dimension has important significance. For the natural image and data subspaces, this variance corresponds to changes in a particular image feature (e.g., animacy). For the ecological and model representational subspaces, however, the variance along a dimension represents how accurately that dimension is encoded by the brain or a particular model. For example, consider Fig 1b in S2. Here, the human brain accurately and precisely represents differences in object animacy (high-variance dimension) but is relatively imprecise in how it represents fine changes in color saturation (low-variance dimension). One way to re-frame this idea is to think of the variance along a given dimension as representing its signal-to-noise-ratio (SNR).

Sampling subspaces Within an image space of ambient dimensionality D_a, we wished to generate multivariate Gaussian subspaces with desired effective dimensionalities and mutual alignment pressures (Fig S3.1 step 1). First, we sampled a natural image subspace $\mathcal{M}_{NI}$ with effective dimensionality ED_{NI}. The orthonormal eigenvectors for this subspace were sampled uniformly within the ambient dimensional space, whereas the eigenvalues were selected deterministically to achieve ED_{NI}. Although there are many ways to design eigenspectra with a particular ED, we opted to parameterize the decay rate of the eigenvalues as a power law $\lambda_i = \frac{1}{i^\alpha}$ and solved for the α that yielded our desired ED.

*Corresponding author.

We next sampled the ecological subspace $\mathcal{M}_{Eco}$, model subspace $\mathcal{M}_{Model}$, and data subspace $\mathcal{M}_{Data}$. To select their eigenvalues, we followed the same power law parameterization as for the natural image subspace to achieve effective dimensionalities ED_{Eco}, ED_{Model}, and ED_{Data}. Their eigenvectors, however, were all sampled in a way that depended on their respective alignment pressures to the natural image subspace $AP_{Eco\leftarrow NI}$, $AP_{Model\leftarrow NI}$, and $AP_{Data\leftarrow NI}$. This aspect of the sampling procedure is described in detail below.

Formulation of AP In our simulations, AP is a scalar value that ranges between -1 and 1. An $AP = 0$ corresponds to no alignment pressure, in which case a basis of eigenvectors is sampled uniformly in the ambient space. When $AP > 0$, eigenvectors are sampled such that dimensions with larger eigenvalues capture more of the total variance in the natural image subspace (i.e., the high-variance dimensions of both subspaces are more likely to be aligned). On the other hand, when $AP < 0$, eigenvectors are sampled to preferentially align with low-variance dimensions of the natural image subspace.

Specifically, we needed to sample orthogonal vectors to form the eigenbasis of a new Gaussian subspace $\mathcal{M}_a$, where those vectors preferentially spanned regions of high-variance in a reference Gaussian subspace $\mathcal{M}_b$, in a way that depended on $AP_{a\leftarrow b}$. We achieved this by first defining a multivariate Gaussian distribution $\mathcal{N}_{a\leftarrow b}(0, \Sigma_{a\leftarrow b})$. The eigenvectors of $\Sigma_{a\leftarrow b}$ were equal to those of $\mathcal{M}_b$, while the eigenvalues of $\Sigma_{a\leftarrow b}$ were generated as follows:

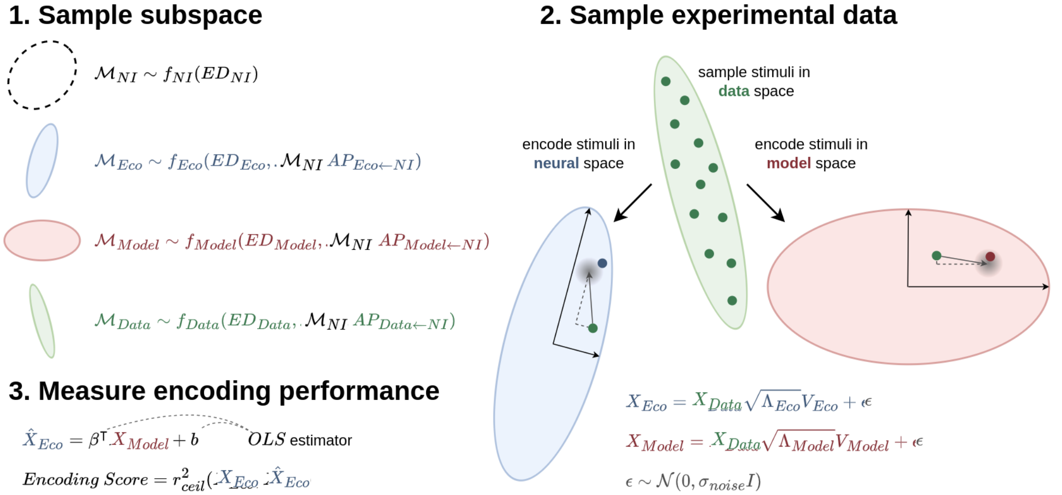

Supplementary Figure S3.1: Simulating our theory of latent dimensionality and encoding performance. 1. Our Gaussian subspaces were sampled to have a desired effective dimensionality. The ecological, model, and data subspaces were also sampled with a given alignment pressure to a shared space: the natural image subspace. **2.** Experimental data were sampled from the distribution specified by the experimental data subspace and then projected onto the ecological and model subspaces, which stretch or compress the data according to their variance along different dimensions. Isotropic noise was then added so that high-variance subspace dimensions had higher SNR and more accurately encoded their corresponding image features. **3.** A linear encoding model was trained using ordinary least squares (OLS) regression to predict neural responses from model activations. The reported encoding score is the percentage of explained variance normalized to the noise-ceiling of the neural data.

$$x_i = \begin{cases} \frac{i-1}{D_a-1}, & \text{if } AP_{a\leftarrow b} \in [0,1] \\ \frac{1-i}{D_a-1}, & \text{if } AP_{a\leftarrow b} \in [-1,0) \end{cases} \quad (1)$$

$$\lambda_i = e^{-sAP_{a\leftarrow b}x_i} \quad (2)$$

where i is the index of the eigenvalue starting from 1, D_a is the ambient dimensionality of the subspaces, and s is a scaling factor which we set to 20. Essentially, this drew eigenvalues from D_a equally spaced points on an exponential function in the domain of [0, 1] that is either decaying (in the case of positive AP) or growing (in the case of negative AP).

Next, we iteratively (1) sampled a vector $v_i \sim \mathcal{N}^{(i)}_{a\leftarrow b}$, (2) normalized v_i to unit length, and (3) projected $\mathcal{N}^{(i)}_{a\leftarrow b}$ onto the subspace orthogonal to v_i, giving $\mathcal{N}^{(i+1)}_{a\leftarrow b}$. This process was repeated D_a times. We then used the normalized v_i's as the eigenvectors of M_a. First, note that the v_i's collectively define an orthonormal basis because each is sampled from a subspace orthogonal to $v_{1:i-1}$. Second, note that for positive $AP_{a\leftarrow b}$ early v_i's are more likely to be oriented towards regions of high variance in M_b, where $\mathcal{N}_{a\leftarrow b}$ has most of its probability mass. For negative $AP_{a\leftarrow b}$, though, the probability mass of $\mathcal{N}_{a\leftarrow b}$ is concentrated along low-variance dimensions in M_b, which results in early v_i's that tend to point in low-variance dimensions of M_b as well. For an $AP_{a\leftarrow b} = 0$, the covariance matrix of $\mathcal{N}_{a\leftarrow b}$ is identity, and the v_i's thus do not depend on the eigenvectors of M_b in any way. Collectively, these properties satisfied our desiderata regarding the function of alignment pressure.

Sampling experimental data Having generated subspaces that specified the distribution of stimuli as well as model and ecological coding properties, our next step was to sample experimental data (Fig S3.1 step 2). First, we sampled N points from the multivariate Gaussian distribution specified by the data subspace:

$$x_i^{Data} \sim \mathcal{N}(0, V_{Data}\Lambda_{Data}V_{Data}^T) \tag{3}$$

where V_{Data} denotes the column matrix of data subspace eigenvectors and Λ_{Data} is the diagonal eigenvalue matrix. These points can be thought of as experimental stimuli, which vary along different image dimensions.

Next, we projected the stimuli onto the eigenvectors of both the ecological and model subspaces (V_{Eco} and V_{Model}) and scaled them by the standard deviation along each of those eigenvectors ($\sqrt{\Lambda_{Eco}}$ and $\sqrt{\Lambda_{Model}}$). The net effect of this scaling was that the ecological/model subspaces amplified or attenuated different stimulus dimensions depending on whether or not they had significant variance along them. However, only applying this scaling would have had no effect on linear encoding performance, since regression weights could re-scale to compensate. Therefore, after performing this projection and scaling, we added ambient noise $\epsilon \sim \mathcal{N}(0, \sigma_{noise} I)$ across all dimensions. The final result was a dataset of neural and model activations:

$$X_{Eco} = X_{Data}\sqrt{\Lambda_{Eco}}V_{Eco} + \epsilon \tag{4}$$

$$X_{Model} = X_{Data}\sqrt{\Lambda_{Model}}V_{Model} + \epsilon \tag{5}$$

Since the magnitude of the noise was equal in all directions, its net effect was to modulate the SNR along the subspace dimensions. Essentially, ecological/model dimensions with high variance were relatively unaffected by the noise and accurately encoded stimulus features, whereas dimensions with low variance were dominated by the noise and only coarsely encoded stimulus features (e.g., the ordering of different stimuli along noise-dominated dimensions might not be preserved).

Measuring encoding performance After having stimulated neural and model activations, our last step was to measure the linear encoding performance of predicting X_{Eco} from X_{Model}. Specifically, we predicted neural activations $\hat{X}_{Eco}$ and then computed the percentage of explained variance normalized to the noise ceiling of X_{Eco}:

$$\hat{X}_{Eco} = \beta X_{Model} + b \tag{6}$$

$$Encoding\ Score = r^2_{ceil}(X_{Eco}, \hat{X}_{Eco}) \tag{7}$$

where the regression parameters β and b were estimated using ordinary least squares regression without any regularization and prediction accuracy was computed through cross-validation. The noise ceiling corresponded to the percentage of variance in X_{Eco} that was explainable signal. In our simulations, we had direct access to this value because E_{Eco} was generated according to:

$$
\begin{aligned}
X_{Eco} &= X_{Data}\sqrt{\Lambda_{Eco}}V_{Eco} + \epsilon \quad (8)\\
&= X_{Eco}^{(signal)} + \epsilon \quad (9)
\end{aligned}
$$

where $X_{Eco}^{(signal)}$ is the signal component of X_{Eco}. Thus, we simply fit another linear regression model to predict X_{Eco} using $X_{Eco}^{(signal)}$ as regressors, in which case the resulting percentage of explained variance r^2 corresponded to the noise ceiling.

When computing percentages of explained variance, we also needed to aggregate across all dimensions (i.e., neurons) of X_{Eco} that were predicted. Typically, this is done by taking the mean r_i^2 across all dimensions i, but this would violate an important principal of our theory wherein dimensions with larger variance contain more signal, and are therefore more important to predict. Instead, we computed a weighted average of all r_i^2, with weights equal to the variance in X_{Eco} along dimension i.

Simulation parameters Unless otherwise stated, our simulation parameters were set as follows: $D_a = 100$, $ED_{NI} = 20$, $ED_{Eco} = 10$, $ED_{Data} = 100$, $AP_{Eco \leftarrow NI} = 0.75$, $AP_{Model \leftarrow NI} = 0.75$, $\sigma_{noise} = 0.1$. ED_{Model} ranged from 1 to D_a, and 50 repeats of the simulation were performed for all values of ED_{Model}, each with independently sampled subspaces/datasets.
