# Supplementary material for: High-performing neural network models of visual cortex benefit from high latent dimensionality
Source: PLoS Comput Biol. 2024 Jan 10;20(1):e1011792. doi: 10.1371/journal.pcbi.1011792 (PMC10805290; doi:10.1371/journal.pcbi.1011792)
Supplement: S4 Text — Results for additional analyses that we conducted by varying parameters of the simulations described in Section Dimensionality and alignment in computational brain models. (PDF) [file pcbi.1011792.s004.pdf]

High-performing neural network models of visual cortex benefit from high latent dimensionality

Eric Elmoznino*
Department of Cognitive Science
Johns Hopkins University
Baltimore, MD 21218
eric.elmoznino@gmail.com

Michael F. Bonner
Department of Cognitive Science
Johns Hopkins University
Baltimore, MD 21218
mfbonner@jhu.edu

S4 - Additional simulation results

In this section, we show how the relationship between ED_{Model} and encoding performance can be modulated by different settings of ED_{Eco}, AP, and σ_{noise}. Fig S4.1 demonstrates these effects, which we interpret below.

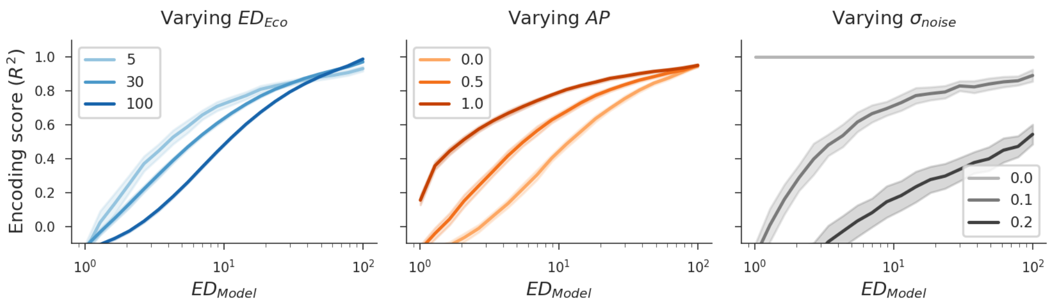

Supplementary Figure S4.1: Modulating additional simulation parameters. Each simulation parameter modulated the relationship between ED_{Model} and encoding performance. Within a plot, only the titled parameter was changed (shown in different line colors), while other parameters were held constant.

Increasing ED_{Eco} As ED_{Eco} increased, higher ED_{Model} was needed to achieve the same level of encoding performance simply because there were more ecological dimensions to explain. In practice, this means that if the ecological subspace (i.e., representations in visual cortex) is high-dimensional, encoding performance will saturate later as a function of ED_{Model}.

Increasing AP In regards to different AP between the model and ecological subspaces to the natural image subspace, we see that lower-dimensional models were able to achieve better encoding performance if they were preferentially aligned to ecological dimensions. Nevertheless, given any constant alignment pressure, there remains a positive correlation between ED_{Model} and encoding performance, indicating independent contributions.

Increasing σ_{noise} Varying σ_{noise} is key to simulations of our theory. In the case of no noise ($\sigma_{noise} = 0$), encoding performance was in fact independent of ED_{Model}, and all models achieved perfect encoding performance. This is because our models had *some* non-zero variance along every ambient dimension, which, in the absence of noise, always led to an SNR of ∞. In essence, the variance along a dimension had no semantic meaning in this case because it could be scaled arbitrarily without any change in the representation. As σ_{noise} increased, however, having high variance along a

*Corresponding author.

dimension became increasingly more important for accurately representing features with high SNR, and encoding performance therefore became more dependent on high ED_{Model}.

Limitations of our theory and simulations While our simulations provide valuable intuitions regarding ED, AP, and encoding performance, they make several simplifying assumptions that are unlikely to hold in practice. First, they assume that all subspaces are multivariate Gaussians, in which case linear metrics such as ED are appropriate for estimating latent dimensionality. While the precise topologies of subspaces in biological and artificial neural representations are unknown, there is evidence that they are likely nonlinear [1]. Another simplification is that our simulations sample linear model and neural dimensions within the *same* ambient space, whereas in reality models and the brain both nonlinearly transform image dimensions. In other words, an image feature that is linearly encoded in the ecological subspace might be highly curved and warped in the model subspace. Future work could build on our simulation framework to explore these issues.

References

[1] Alessio Ansuini, Alessandro Laio, Jakob H. Macke, and Davide Zoccolan. Intrinsic dimension of data representations in deep neural networks. *CoRR*, abs/1905.12784, 2019. URL `http://arxiv.org/abs/1905.12784`.
