# Supplementary material for: High-performing neural network models of visual cortex benefit from high latent dimensionality
Source: PLoS Comput Biol. 2024 Jan 10;20(1):e1011792. doi: 10.1371/journal.pcbi.1011792 (PMC10805290; doi:10.1371/journal.pcbi.1011792)
Supplement: S5 Text — Descriptions of all DNN models used in our analyses. (PDF) [file pcbi.1011792.s005.pdf]

High-performing neural network models of visual cortex benefit from high latent dimensionality

Eric Elmoznino*
Department of Cognitive Science
Johns Hopkins University
Baltimore, MD 21218
eric.elmoznino@gmail.com

Michael F. Bonner
Department of Cognitive Science
Johns Hopkins University
Baltimore, MD 21218
mfbonner@jhu.edu

S5 - Details of DNN models

Table S5.1 lists details for all DNNs used in our experiments. *PyTorch* models were obtained from the torchvision package and from PyTorch Hub [1], *VVS* models were obtained from Zhuang et al. [3], and *Taskonomy* models were obtained from Zamir et al. [2].

*Corresponding author.

Table S5.1: DNN models used in experiments

Training task	Learning setting	Training dataset	Architecture	Source
Object classification	Supervised	ImageNet	AlexNet	PyTorch
Object classification	Supervised	ImageNet	VGG-16	PyTorch
Object classification	Supervised	ImageNet	SqueezeNet	PyTorch
Object classification	Supervised	ImageNet	ResNet18	PyTorch
Object classification	Supervised	ImageNet	ResNet50	PyTorch
Barlow-Twins	Self-Supervised	ImageNet	ResNet50	PyTorch
N/A	Untrained	N/A	AlexNet	N/A
N/A	Untrained	N/A	VGG-16	N/A
N/A	Untrained	N/A	SqueezeNet	N/A
N/A	Untrained	N/A	ResNet18	N/A
N/A	Untrained	N/A	ResNet50	N/A
Object classification	Supervised	ImageNet	ResNet18	VVS
Depth prediction	Supervised	ImageNet	ResNet18	VVS
Auto-encoding	Self-supervised	ImageNet	ResNet18	VVS
Colorization	Self-supervised	ImageNet	ResNet18	VVS
Contrastive multiview coding	Self-supervised	ImageNet	ResNet18	VVS
Contrastive predictive coding	Self-supervised	ImageNet	ResNet18	VVS
Deep cluster	Self-supervised	ImageNet	ResNet18	VVS
Instance recognition	Self-supervised	ImageNet	ResNet18	VVS
Local aggregation	Self-supervised	ImageNet	ResNet18	VVS
Relative position	Self-supervised	ImageNet	ResNet18	VVS
SimCLR	Self-supervised	ImageNet	ResNet18	VVS
Object classification	Supervised	Indoor buildings	ResNet50	Taskonomy
Scene classification	Supervised	Indoor buildings	ResNet50	Taskonomy
Semantic segmentation	Supervised	Indoor buildings	ResNet50	Taskonomy
Curvature estimation	Supervised	Indoor buildings	ResNet50	Taskonomy
Depth estimation	Supervised	Indoor buildings	ResNet50	Taskonomy
Depth estimation (z-buffer)	Supervised	Indoor buildings	ResNet50	Taskonomy
Edge detection (2D)	Supervised	Indoor buildings	ResNet50	Taskonomy
Edge detection (3D)	Supervised	Indoor buildings	ResNet50	Taskonomy
Egomotion	Supervised	Indoor buildings	ResNet50	Taskonomy
Fixated pose estimation	Supervised	Indoor buildings	ResNet50	Taskonomy
Non-fixated pose estimation	Supervised	Indoor buildings	ResNet50	Taskonomy
Keypoint detection (2D)	Supervised	Indoor buildings	ResNet50	Taskonomy
Keypoint detection (3D)	Supervised	Indoor buildings	ResNet50	Taskonomy
Point matching	Supervised	Indoor buildings	ResNet50	Taskonomy
Reshading	Supervised	Indoor buildings	ResNet50	Taskonomy
Room layout estimation	Supervised	Indoor buildings	ResNet50	Taskonomy
Surface normal estimation	Supervised	Indoor buildings	ResNet50	Taskonomy
Vanishing point estimation	Supervised	Indoor buildings	ResNet50	Taskonomy
Auto-encoding	Self-supervised	Indoor buildings	ResNet50	Taskonomy
Denoising	Self-supervised	Indoor buildings	ResNet50	Taskonomy
Inpainting	Self-supervised	Indoor buildings	ResNet50	Taskonomy
Jigsaw	Self-supervised	Indoor buildings	ResNet50	Taskonomy
Unsupervised segmentation (2D)	Self-supervised	Indoor buildings	ResNet50	Taskonomy
Unsupervised segmentation (2.5D)	Self-supervised	Indoor buildings	ResNet50	Taskonomy

References

[1] Adam Paszke, Sam Gross, Francisco Massa, Adam Lerer, James Bradbury, Gregory Chanan, Trevor Killeen, Zeming Lin, Natalia Gimelshein, Luca Antiga, Alban Desmaison, Andreas Kopf, Edward Yang, Zachary DeVito, Martin Raison, Alykhan Tejani, Sasank Chilamkurthy, Benoit Steiner, Lu Fang, Junjie Bai, and Soumith Chintala. Pytorch: An imperative style, high-performance deep learning library. In H. Wallach, H. Larochelle, A. Beygelzimer, F. d'Alché-Buc, E. Fox, and R. Garnett, editors, *Advances in Neural Information Processing Systems 32*, pages 8024–8035. Curran Associates, Inc., 2019. URL `http://papers.neurips.cc/paper/9015-pytorch-an-imperative-style-high-performance-deep-learning-library.pdf`.

[2] Amir R Zamir, Alexander Sax, William B Shen, Leonidas Guibas, Jitendra Malik, and Silvio Savarese. Taskonomy: Disentangling task transfer learning. In *2018 IEEE Conference on Computer Vision and Pattern Recognition (CVPR)*. IEEE, 2018.

[3] Chengxu Zhuang, Siming Yan, Aran Nayebi, Martin Schrimpf, Michael C. Frank, James J. DiCarlo, and Daniel L. K. Yamins. Unsupervised neural network models of the ventral visual stream. *Proceedings of the National Academy of Sciences*, 118(3), 2021. ISSN 0027-8424. doi: 10.1073/pnas.2014196118. URL `https://www.pnas.org/content/118/3/e2014196118`.
