# Supplementary material for: High-performing neural network models of visual cortex benefit from high latent dimensionality
Source: PLoS Comput Biol. 2024 Jan 10;20(1):e1011792. doi: 10.1371/journal.pcbi.1011792 (PMC10805290; doi:10.1371/journal.pcbi.1011792)
Supplement: S6 Text — Analyses that examine the relationship between ED and encoding performance under different conditions from those described in the main paper. (PDF) [file pcbi.1011792.s006.pdf]

High-performing neural network models of visual cortex benefit from high latent dimensionality

Eric Elmoznino*
Department of Cognitive Science
Johns Hopkins University
Baltimore, MD 21218
eric.elmoznino@gmail.com

Michael F. Bonner
Department of Cognitive Science
Johns Hopkins University
Baltimore, MD 21218
mfbonner@jhu.edu

S6 - Additional analyses of ED and encoding performance

Here, we replicate our main results in more settings. Fig S6.1 shows the relationship between effective dimensionality and encoding performance without applying an average-pooling operation to the DNN feature maps. Fig S6.2 colors models according to what dataset they were pre-trained on (ImageNet or Taskonomy). Figures S6.3 and S6.4 show encoding performance across multiple species, recording modalities, and brain regions. Fig S6.5 fits encoding models using OLS instead of partial-least-squares regression. Fig S6.6 shows results for only the layer of each model that achieved the highest encoding performance, rather than all layers.

Our general results hold across all of these settings, with the exception of V1 in the monkey electrophysiology data. We speculate that this is due to the far lower complexity of representations in V1, which serve primarily as simple edge detectors for later processing in higher-level regions.

*Corresponding author.

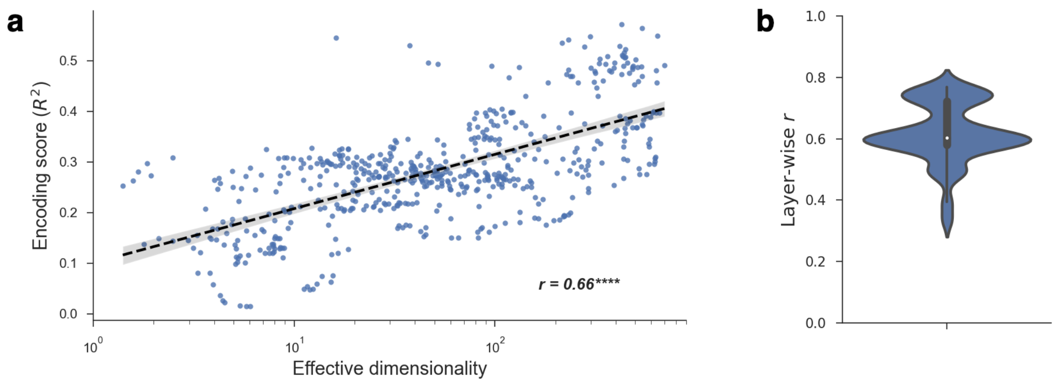

Supplementary Figure S6.1: Effective dimensionality and encoding performance without average-pooling. a. The encoding performance achieved by a model scaled with the effective dimensionality of its entire feature map (without average-pooling applied). Each point in the plot was obtained from one layer from one DNN, resulting in a total of 568 models (see main text for further details). **b.** Even when conditioning on a particular DNN layer, controlling for both depth and ambient dimensionality, effective dimensionality and encoding performance continued to strongly correlate. The plot shows the distribution of these correlations (Pearson r) across all unique layers in our analyses.

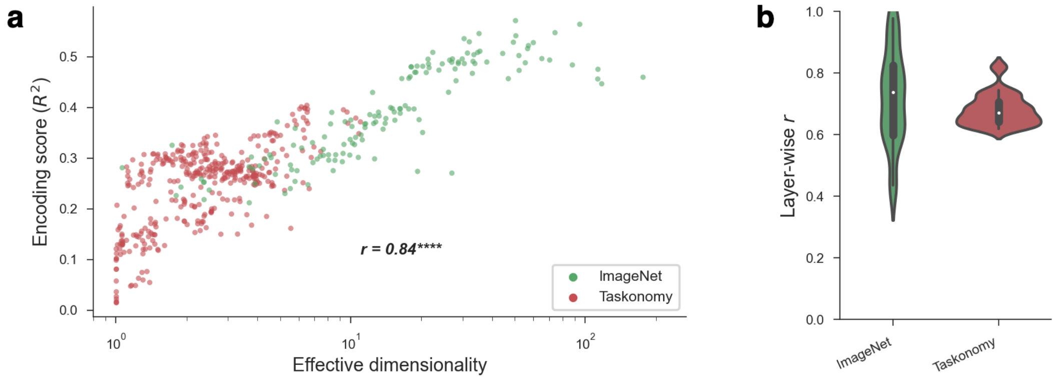

Supplementary Figure S6.2: Effective dimensionality and encoding performance by training set. a. The encoding performance achieved by a model scaled with the effective dimensionality of its features, within both ImageNet- and Taskonomy-trained models. **b.** Even when conditioning on a particular DNN layer, controlling for both depth and ambient dimensionality, effective dimensionality and encoding performance continued to strongly correlate for both training sets. The plot shows the distribution of these correlations (Pearson r) across all unique layers in our analyses.

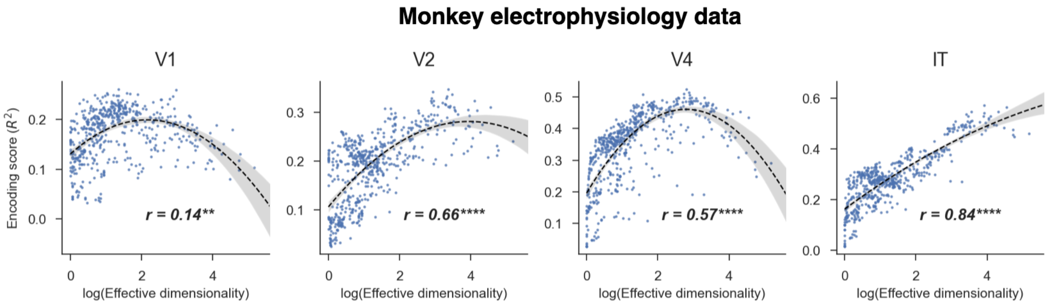

Supplementary Figure S6.3: Latent dimensionality and encoding performance on monkey electrophysiology data. The encoding performance for all of our models across multiple brain regions in monkey electrophysiology datasets collected by Majaj et al. [3] (IT and V4) and Freeman et al. [2] (V1 and V2), plotted against the models' ED. Our results hold across all brain regions except for V1; encoding performance increases with latent dimensionality. Another possible interpretation of these results, which becomes apparent when fitting inverted-U curves to the data (2nd-order polynomial regression), is that each region (including V1) has an "optimal" model ED reminiscent of the "Joint regime" illustrated in Fig 1c of our main manuscript, which tends to increase along the cortical hierarchy. Note that optimal model ED values can only be compared within (V1, V2) and (V4, IT), since recordings for each set of regions were obtained from different stimulus sets.

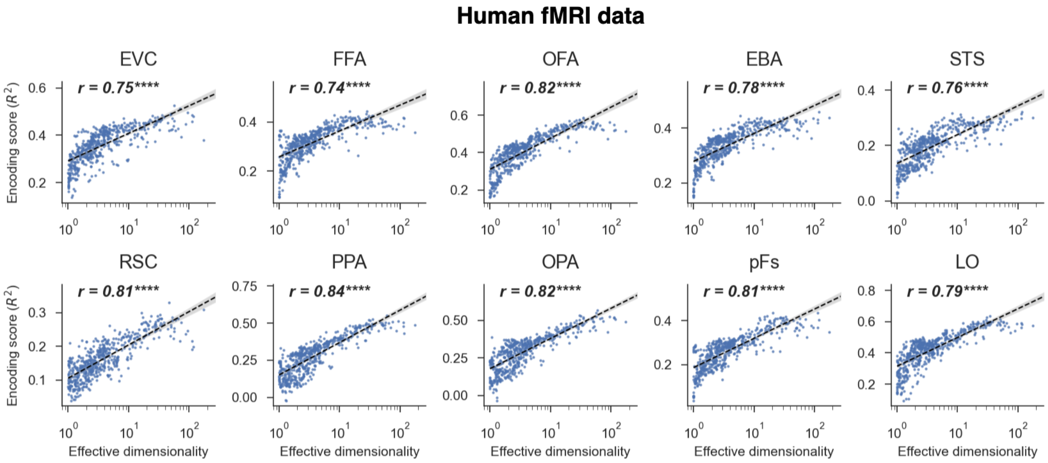

Supplementary Figure S6.4: Latent dimensionality and encoding performance on human fMRI data. The encoding performance for all of our models across multiple brain regions in a human fMRI dataset collected by Bonner and Epstein [1], plotted against the models' ED. Our results hold across all brain regions; encoding performance increases with latent dimensionality. EVC=early visual cotex, FFA=fusiform face area, OFA=occipital face area, EBA=extrastriate body area, RSC=retrosplenial complex, PPA=parahippocampal place area, OPA=occipital place area, STS-superior temporal sulcus, LO=lateral occipital region, pFs=posterior fusiform region.

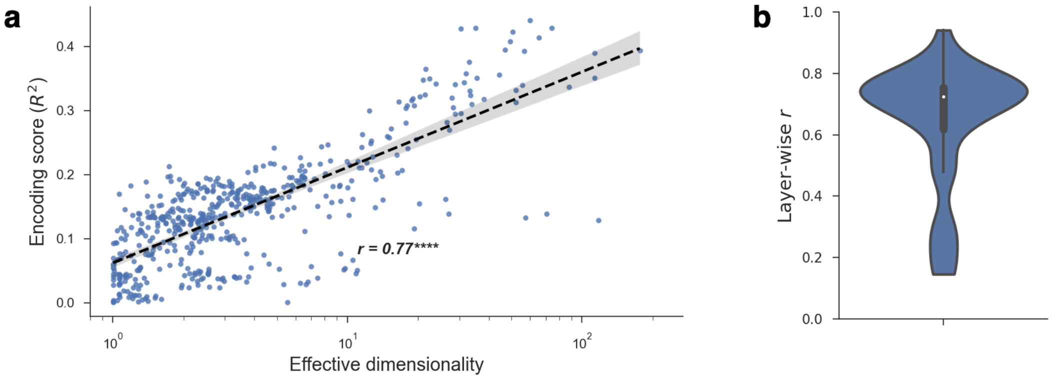

Supplementary Figure S6.5: Effective dimensionality and encoding performance using OLS regression. a. The encoding performance achieved by a model fit using OLS regression scaled with its effective dimensionality. Each point in the plot was obtained from one layer from one DNN, resulting in a total of 568 models (see main text for further details). **b.** Even when conditioning on a particular DNN layer, controlling for both depth and ambient dimensionality, effective dimensionality and encoding performance continued to strongly correlate. The plot shows the distribution of these correlations (Pearson r) across all unique layers in our analyses.

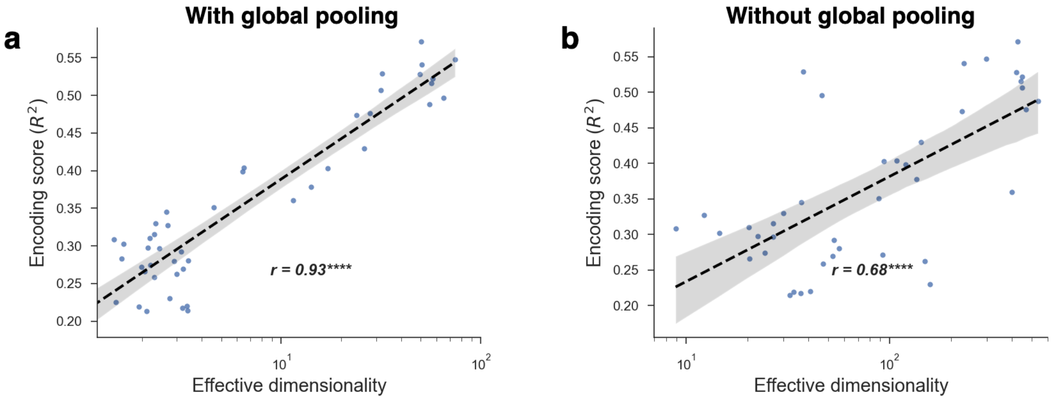

Supplementary Figure S6.6: Effective dimensionality and encoding performance using the best layer from each model. Instead of showing results for all layers of each DNN, an alternative method is to consider only the layer that achieves the best encoding performance as the "model". Even using this approach, there is still a clear trend between model ED and encoding performance. **a.** With global pooling applied to the model features prior to computing ED. **b.** Without global pooling applied.

References

[1] Michael F. Bonner and Russell A. Epstein. Object representations in the human brain reflect the co-occurrence statistics of vision and language. *Nature Communications*, 12(1):4081, Jul 2021. ISSN 2041-1723. doi: 10.1038/s41467-021-24368-2. URL `https://doi.org/10.1038/s41467-021-24368-2`.

[2] Jeremy Freeman, Corey M Ziemba, David J Heeger, Eero P Simoncelli, and J Anthony Movshon. A functional and perceptual signature of the second visual area in primates. *Nature neuroscience*, 16(7): 974–981, 2013.

[3] Najib J. Majaj, Ha Hong, Ethan A. Solomon, and James J. DiCarlo. Simple learned weighted sums of inferior temporal neuronal firing rates accurately predict human core object recognition performance. *Journal of Neuroscience*, 35(39):13402–13418, 2015. ISSN 0270-6474. doi: 10.1523/JNEUROSCI.5181-14.2015. URL `https://www.jneurosci.org/content/35/39/13402`.
