# Supplementary material for: High-performing neural network models of visual cortex benefit from high latent dimensionality
Source: PLoS Comput Biol. 2024 Jan 10;20(1):e1011792. doi: 10.1371/journal.pcbi.1011792 (PMC10805290; doi:10.1371/journal.pcbi.1011792)
Supplement: S7 Text — Comparing ED to neural data using representational similarity analysis instead of encoding performance. (PDF) [file pcbi.1011792.s007.pdf]

High-performing neural network models of visual cortex benefit from high latent dimensionality

Eric Elmoznino*
Department of Cognitive Science
Johns Hopkins University
Baltimore, MD 21218
eric.elmoznino@gmail.com

Michael F. Bonner
Department of Cognitive Science
Johns Hopkins University
Baltimore, MD 21218
mfbonner@jhu.edu

S7 - ED and Representational Similarity Analysis

To provide additional evidence that our central results are not due to trivial statistical effects wherein models with higher latent dimensionality have more degrees of freedom to predict neural data, we replicated our results using representational similarity analysis (RSA) [1]. In RSA, a dissimilarity matrix is constructed for both the model and the brain data by computing a distance between the representations for each pair of stimuli. These matrices are then correlated to evaluate their similarity. Importantly, unlike when fitting encoding models, this method for measuring the similarity between a model and the brain is entirely non-parametric and thus cannot be biased to favour models with high latent dimensionality.

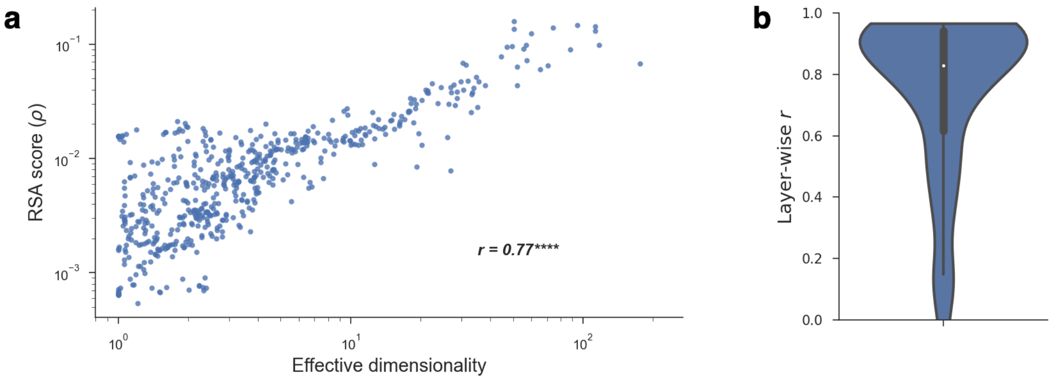

Supplementary Figure S7.1: Effective dimensionality and Representational Similarity Analysis (RSA). We compared the representations in our models to those in the monkey IT electrophysiology data using RSA. Note that the y-axis is on a log-scale in order to provide better resolution in face of the high variation in RSA scores. Our results hold across this different similarity metric; the similarity between model and brain representational dissimilarity matrices increases with latent dimensionality.

While not as strong, there is nevertheless a clear trend in which models with higher RSA scores tend to have higher ED. Thus, our core results replicate using RSA.

References

[1] Nikolaus Kriegeskorte, Marieke Mur, and Peter Bandettini. Representational similarity analysis - connecting the branches of systems neuroscience. *Frontiers in Systems Neuroscience*, 2, 2008. ISSN 1662-5137. doi: 10.3389/neuro.06.004.2008. URL https://www.frontiersin.org/article/10.3389/neuro.06.004.2008.

*Corresponding author.
