# Supplementary material for: High-performing neural network models of visual cortex benefit from high latent dimensionality
Source: PLoS Comput Biol. 2024 Jan 10;20(1):e1011792. doi: 10.1371/journal.pcbi.1011792 (PMC10805290; doi:10.1371/journal.pcbi.1011792)
Supplement: S8 Text — Trends in ED across factors of variation in our models, such as layer depth and pre-training dataset. (PDF) [file pcbi.1011792.s008.pdf]

High-performing neural network models of visual cortex benefit from high latent dimensionality

Eric Elmoznino*
Department of Cognitive Science
Johns Hopkins University
Baltimore, MD 21218
eric.elmoznino@gmail.com

Michael F. Bonner
Department of Cognitive Science
Johns Hopkins University
Baltimore, MD 21218
mfbonner@jhu.edu

S8 - ED varies with model and training parameters

To better understand how and why latent dimensionality varies in DNNs (and perhaps manipulate it for a desired effect), we can start by observing its empirical relationship to parameters of the training procedure, dataset, and architecture. Fig S8.1 illustrates several of these relationships, which we were able to quantify thanks to the use of our large bank of models. We summarize our most important conclusions from these analyses below.

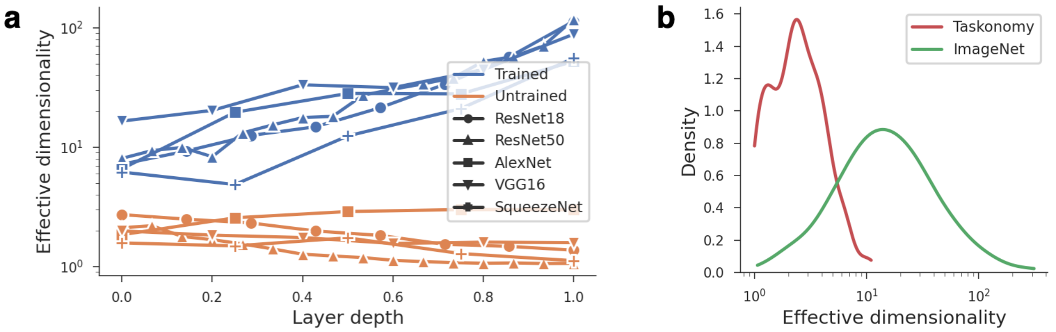

Supplementary Figure S8.1: Effective dimensionality varies with model and training parameters. a. Models trained on object classification (blue) had larger effective dimensionality than untrained models (orange) across layers in multiple architectures. After training, effective dimensionality also gradually increased as a function of layer depth (only convolutional layers are shown). **b.** Plots indicate the distribution of effective dimensionality across models that were trained on Taskonomy (red) and ImageNet (green). These distributions differed significantly, despite the models in both groups largely sharing similar architectures and training tasks.

Training increases effective dimensionality Fig S8.1a shows how effective dimensionality varied across the layer hierarchy for multiple CNN architectures when they were trained on ImageNet object classification compared to when they were untrained and had randomly initialized weights. We can see that training resulted in substantial increases in effective dimensionality for both architectures across all layers. To solve complex tasks such as object classification, then, it appears that models must learn to extract a large number of orthogonal image features. This finding contradicts a commonly held belief that DNNs trained on visual tasks compress high-dimensional inputs to a small number of latent dimensions [6, 2, 5, 1, 4].

Effective dimensionality increases with layer depth Another notable trend in Fig S8.1a is that effective dimensionality increased as a function of layer depth within the two supervised classification

*Corresponding author.

models we considered. Importantly, this cannot be explained simply as a result of an increasing number of channels along the layer hierarchy, as effective dimensionality remained more or less constant within the untrained models. This gradual increase in effective dimensionality appears to contradict other findings from [1, 3, 2] in which latent dimensionality generally decreases as a function of layer depth, but there are important methodological differences to note. First and foremost, we computed effective dimensionality only along the channel dimension of our feature maps after applying a average-pooling operation across the spatial dimensions, which allowed us to focus on the diversity of image features. Given that spatial resolution decreases as a function of layer depth in our architectures (and most convolutional DNNs in general), the effective dimensionality of earlier layers will be higher simply due of the larger number of spatial dimensions that they contain. Another important difference in our work is that we only considered convolutional layers and performed no analyses on fully-connected layers. Indeed, much of the drop in latent dimensionality reported in other work occurs within these fully-connected layers.

Training data has a large impact on effective dimensionality Another important factor that has a significant impact on a model's learned representations is the training dataset. Our bank of models includes DNNs trained on ImageNet and Taskonomy. Fig S8.1b shows the distribution of effective dimensionality for all models trained on each of these datasets. Despite similar architectures and training tasks used on both datasets, the ImageNet-trained models tended to have significantly larger effective dimensionality. Although we did not perform further analyses to determine which dataset differences explain this result, we speculate that it is due to the much greater diversity of image statistics within ImageNet. Whereas ImageNet contains images spanning many object categories appearing in diverse environments, Taskonomy consists solely of man-made indoor scenes across 600 buildings. Effective dimensionality, therefore, might scale in proportion to the complexity and variation of image features in the training data.

References

[1] Alessio Ansuini, Alessandro Laio, Jakob H. Macke, and Davide Zoccolan. Intrinsic dimension of data representations in deep neural networks. *CoRR*, abs/1905.12784, 2019. URL http://arxiv.org/abs/1905.12784.

[2] SueYeon Chung, Daniel D. Lee, and Haim Sompolinsky. Classification and geometry of general perceptual manifolds. *Phys. Rev. X*, 8:031003, Jul 2018. doi: 10.1103/PhysRevX.8.031003. URL https://link.aps.org/doi/10.1103/PhysRevX.8.031003.

[3] Uri Cohen, SueYeon Chung, Daniel D. Lee, and Haim Sompolinsky. Separability and geometry of object manifolds in deep neural networks. *Nature Communications*, 11(1):746, Feb 2020. ISSN 2041-1723. doi: 10.1038/s41467-020-14578-5. URL https://doi.org/10.1038/s41467-020-14578-5.

[4] Ruili Feng, Kecheng Zheng, Yukun Huang, Deli Zhao, Michael Jordan, and Zheng-Jun Zha. Rank diminishing in deep neural networks, 2022. URL https://arxiv.org/abs/2206.06072.

[5] Diederik P Kingma and Max Welling. Auto-encoding variational bayes, 2013. URL https://arxiv.org/abs/1312.6114.

[6] Stefano Recanatesi, Matthew Farrell, Madhu Advani, Timothy Moore, Guillaume Lajoie, and Eric Shea-Brown. Dimensionality compression and expansion in deep neural networks. *CoRR*, abs/1906.00443, 2019. URL http://arxiv.org/abs/1906.00443.
