# Supplementary material for: High-performing neural network models of visual cortex benefit from high latent dimensionality
Source: PLoS Comput Biol. 2024 Jan 10;20(1):e1011792. doi: 10.1371/journal.pcbi.1011792 (PMC10805290; doi:10.1371/journal.pcbi.1011792)
Supplement: S9 Text — Nuanced discussion of the conditions under which our theory predicts that encoding performance will increase with model ED, along with supporting analyses. (PDF) [file pcbi.1011792.s009.pdf]

High-performing neural network models of visual cortex benefit from high latent dimensionality

Eric Elmoznino*
Department of Cognitive Science
Johns Hopkins University
Baltimore, MD 21218
eric.elmoznino@gmail.com

Michael F. Bonner
Department of Cognitive Science
Johns Hopkins University
Baltimore, MD 21218
mfbonner@jhu.edu

S9 - High ED alone is not sufficient to yield strong performance

Our findings show that ED is positively correlated with encoding performance when examining standard DNNs used in computational neuroscience. However, it is important to emphasize that high ED alone is not sufficient to yield strong encoding performance. Indeed, it is not difficult to imagine contrived models with extremely high dimensionality but no predictive power. As a simple example, imagine a maximally sparse representation in which each stimulus elicits a response along a single, unique dimension (akin to "grandmother cells" [1]). In this case, because every stimulus is represented along a unique dimension, an encoding model fit to a training set would have no ability to generalize to unseen stimuli.

In this section, we discuss some of the necessary conditions for observing a strong and positive correlation between effective dimensionality and encoding performance. In addition, we provide some empirical experiments to support our arguments.

Alignment pressure plays a significant independent role In the vocabulary of our theory laid out in Section 2.1 of the main manuscript, encoding performance depends not only on the latent dimensionality of a model, but also on its alignment pressure. And, because latent dimensionality can vary independently from alignment pressure (in the sense of independent causal interventions), it is not causally sufficient for achieving good encoding performance. Furthermore, in the infinite-dimensional space of possible visual features where model dimensions are unlikely to overlap with ecologically-relevant ones by chance, alignment pressure is essential.

In empirical models of visual cortex, the statistical relationship between alignment pressure and latent dimensionality has not been investigated. If models achieving high alignment pressure to biological representations tend to systematically have lower latent dimensionality, we might observe a net negative correlation between latent dimensionality and encoding performance. That this is not the case in our empirical results suggests that alignment pressure is probably uncorrelated (or perhaps positively) correlated with latent dimensionality.

Latent dimensionality must reflect the number of accurately-encoded features If high latent dimensionality improves encoding performance in all circumstances, we should be able to trivially obtain excellent encoding performance with any model by applying simple feature transformations. ZCA whitening, for instance, applies a linear transformation that results in a new set of features with covariance matrix close to identity (i.e., a flat eigenspectrum with maximum effective dimensionality). After applying ZCA to all models, however, we saw no improvement in encoding performance, despite significant increases in effective dimensionality, as shown in Fig S9.1. Again, this shows that effective dimensionality alone is not sufficient to improve encoding performance. In this case, the reason is that ZCA whitening does not augment the model with additional information about the stimulus. Our theory in Section 2.1 of the main manuscript states that the relationship between latent

*Corresponding author.

dimensionality and encoding performance depends on an assumption that higher-variance dimensions more accurately encode stimulus features. ZCA whitening, however, violates this assumption since it increases latent dimensionality by numerically scaling existing model dimensions without changing their semantics. No new dimensions are added and no existing dimensions are encoded more accurately, so encoding performance remains unchanged.

The empirical relationship between dimensionality and encoding performance is robust Despite the above caveats, we note that it is difficult to construct poor-performing high-dimensional models in practice, without having to resort to trivial feature transformations such as whitening. We attempted to do so by training a DNN on a version of ImageNet where the labels in the training set were randomly scrambled. Due to their large capacity, it is well known that DNNs are able to achieve low training error on this task by finding an arbitrary mapping between each input and its label, essentially memorizing the dataset [3]. Our rationale for choosing this task was that it is unlikely to produce ecologically-relevant dimensions, but stands a good chance of learning a high-dimensional latent space in which it is easier to linearly separate arbitrarily labeled data [2]. However, this turned out *not* to be the case. In Fig S9.2, we show the effective dimensionality and encoding performance of a DNN fit to scrambled labels and compare it to an identical architecture fit with the correct labels. As expected, the DNN with scrambled labels achieved much lower encoding performance. Surprisingly, however, this model also had much lower effective dimensionality than its correctly trained counterpart. We speculate that ecologically-relevant visual tasks in which humans excel (and most DNNs are trained on) require high latent dimensionality as a result of their inherent complexity, producing a positive correlation between latent dimensionality and alignment pressure. We explore this possibility in Section 2.4 of the main manuscript.

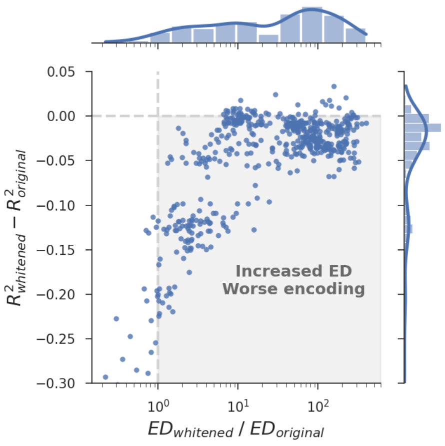

Supplementary Figure S9.1: Increasing latent dimensionality with ZCA whitening does not enhance encoding performance. The y-axis shows the difference in encoding performance after whitening model features, while the x-axis shows the ratio of increase in effective dimensionality. Most whitened models saw a substantial increase in effective dimensionality, but showed either no change in encoding performance or a decrease (highlighted gray region).

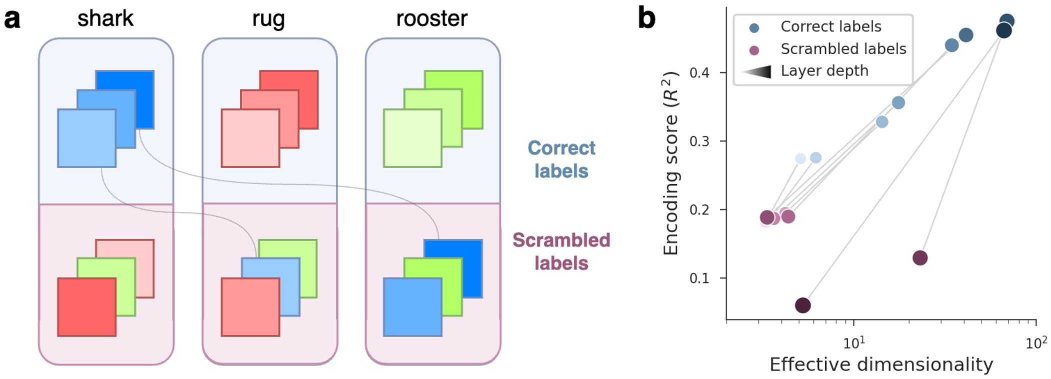

Supplementary Figure S9.2: Training a model to overfit scrambled labels does not increase latent dimensionality. a. We trained the same ResNet18 DNN architecture on ImageNet classification in two different settings: once with correctly labeled images (blue) and the other time with scrambled labels, such that each image was assigned to a random class. Despite the arbitrary nature of the second task, the model was able to achieve good performance on the training set (47% accuracy over 1000 classes) by memorizing an idiosyncratic mapping from each input to its label. **b.** Our initial hypothesis was that the model trained with scrambled labels would have higher effective dimensionality and lower encoding performance than the model trained with correct labels, but our results surprisingly run counter to this intuition: the model trained with scrambled labels had lower encoding performance and *lower* effective dimensionality. Blue points denote layers from the model trained with correct labels, and purple points denote layers from the model with scrambled labels. Size and brightness denote increasing layer depth, and lines indicate matching layers.

References

[1] Ann-Sophie Barwich. The value of failure in science: The story of grandmother cells in neuroscience. *Frontiers in neuroscience*, 13:1121–1121, Oct 2019. ISSN 1662-4548. doi: 10.3389/fnins.2019.01121. URL `https://doi.org/10.3389/fnins.2019.01121`. 31708726[pmid].

[2] Alexander N Gorban, Valery A Makarov, and Ivan Y Tyukin. High-Dimensional brain in a High-Dimensional world: Blessing of dimensionality. *Entropy (Basel)*, 22(1), January 2020.

[3] Chiyuan Zhang, Samy Bengio, Moritz Hardt, Benjamin Recht, and Oriol Vinyals. Understanding deep learning requires rethinking generalization. *CoRR*, abs/1611.03530, 2016. URL `http://arxiv.org/abs/1611.03530`.
