# Supplementary material for: High-performing neural network models of visual cortex benefit from high latent dimensionality
Source: PLoS Comput Biol. 2024 Jan 10;20(1):e1011792. doi: 10.1371/journal.pcbi.1011792 (PMC10805290; doi:10.1371/journal.pcbi.1011792)
Supplement: S10 Text — Comparison of encoding performance to DNN features other than ED, such as classification performance and other geometric statistics of the representations. (PDF) [file pcbi.1011792.s010.pdf]

High-performing neural network models of visual cortex benefit from high latent dimensionality

Eric Elmoznino*
Department of Cognitive Science
Johns Hopkins University
Baltimore, MD 21218
eric.elmoznino@gmail.com

Michael F. Bonner
Department of Cognitive Science
Johns Hopkins University
Baltimore, MD 21218
mfbonner@jhu.edu

S10 - Other correlates of DNN encoding model performance

In this section, we directly address alternate hypotheses proposed in the literature to explain the performance of DNN models of visual cortex. While these can be cast as competing hypotheses to our proposed theory based on ED, we will argue that task-performance measures and geometric properties should be instead viewed as different levels of explanation that provide complementary views of the function and underlying structure of neural representations.

Object classification performance A commonly held view is that DNNs explain neural data because their representations are optimized to perform well on ethologically-relevant tasks, such as object classification [7, 6, 2]. Indeed, in our models, we found that few-shot classification performance on object categories from ImageNet-21k predicted encoding performance very well (Fig S10.1a). However, it is important to note that task performance and ED are not competing theories but, rather, different levels of explanation that provide complementary insights into the nature of neural network representations. Models that perform ethologically-relevant visual tasks are likely to explain neural responses, because the brain is optimized for such tasks as well and the space of good solutions is likely limited. However, this does not tell us *how* the models solve these tasks, for which we must look at the underlying representational structure. In the language of Marr [3], theories based on classification performance function at the abstract computational level, but there must also be explanations for DNN encoding performance that function at the representational level. ED is one such representation-based explanation. In Sections 2.4 and 2.5 of the main manuscript, we showed how ED empirically predicts classification performance on novel categories and provided one theoretical explanation for why this happens, which was proposed by Sorscher et al. [5]. Furthermore, when we conducted a variance partitioning analysis [4, 1] of encoding performance as a function of both ED and classification performance (Fig S10.1b), we found that nearly all of the variance was shared. In sum, there is good reason to believe that ED and classification performance (or visual task performance more generally) are causally intertwined, and that they jointly provide a richer explanation of DNN models of visual cortex.

Other geometric properties of the representations To compare our theory to other representation-based hypotheses, we considered several geometric properties of representations that were described in Sorscher et al. [5] to predict the few-shot classification performance of pre-trained DNNs. For instance, one such geometric property is *signal*, which quantifies the average distance between pairs of class centroids in the representation. Some of these properties also relate to ED, except that they are computed either per object category and then averaged ("within-concept ED"), or they are computed using object category centroids ("between-concept ED"). Finally, the signal-to-noise ratio property (SNR) is an aggregate metric that combines all of the others to predict few-shot classification performance on novel categories using a prototype learning rule. See Sorscher et al. [5] and their related code (or ours) for a more complete description of these geometric properties and how they

*Corresponding author.

were computed. We used the same setup, with the same ImageNet-21k categories as Sorscher et al. The one exception is "between-concept ED", which was not computed in Sorscher et al. [5], and simply consists of computing ED using object category centroids as the samples. The relationships between all of these geometric properties and encoding performance are shown in Fig S10.1c.

We found that ED-related properties of the representation (i.e., global, within-concept, and between-concept ED) have the strongest relationship to encoding performance, with the exception of the aggregate SNR metric. This shows that among several geometric statistics of a representation, a simple measure of its latent dimensionality can most accurately predict its performance as a model of higher visual cortex. We wondered whether even the performance of SNR—a more complex aggregate metric that incorporates ED—could largely be accounted for by ED as well. We therefore conducted variance partitioning analyses of both encoding performance and classification performance on ImageNet-21k using ED and SNR as the predictors (Fig S10.1d). Indeed, we found that almost all of the variance is shared, which suggests that ED is the primary driver of high SNR. We then conducted similar variance partitioning analyses using ED and "signal" as the predictors, since "signal" was the metric that best predicted encoding performance among non-ED metrics. Surprisingly, we found that ED explained significant unique variance, while "signal" did not. It therefore appears that "signal" only explains encoding and classification performance insofar as it correlates with ED, whereas ED explains unique variance in model performance.

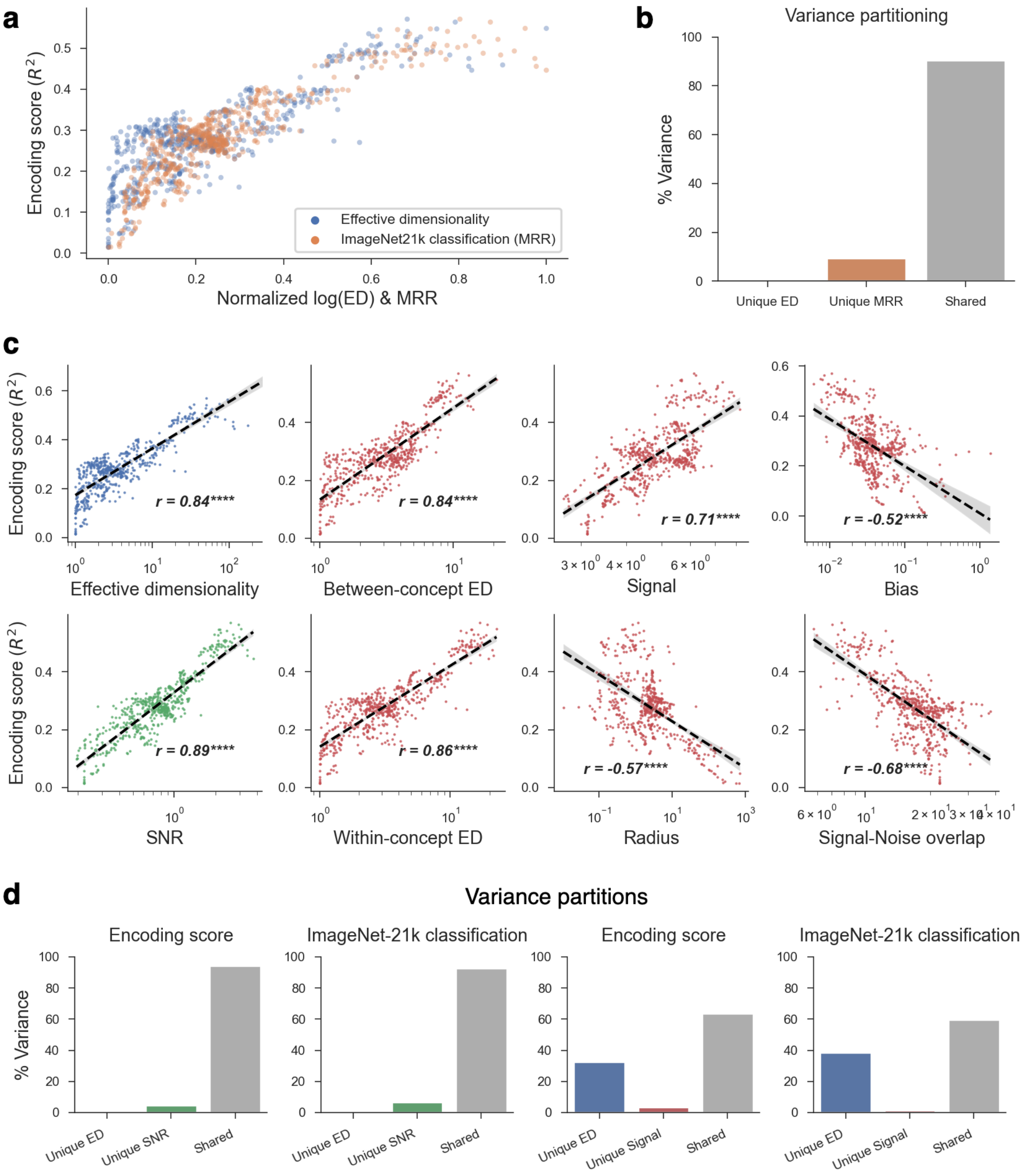

Supplementary Figure S10.1: A theory of encoding performance based on ED is compatible with one based on classification performance, and superior to one based on other geometric properties of a representation. **a.** Encoding performance vs. ED (blue) and ImageNet-21k classification performance quantified using the mean reciprocal rank (MRR, orange). Both metrics were highly predictive of encoding performance. Note that the x-axis shows both metrics normalized to be between (0-1) so that they can easily be compared in the same plot. **b.** A variance partitioning analysis showed that ED and classification performance explain almost no unique variance in encoding performance, with about 90% of it being shared. This suggests that ED and classification performance should not be considered as independent and competing hypotheses, but are rather different levels of explanation that provide a complementary perspective. **c.** Encoding performance vs. ED (blue) and other geometric properties of a representation introduced in Sorscher et al. [5] (red for most properties, green for SNR—an aggregate property computed from the others). Aside from SNR, ED-related metrics had the strongest relationship to encoding performance. **d.** We partitioned the variance explained in both encoding performance and classification performance by ED, SNR, and "signal", which is another key geometric property. SNR shares almost all of its variance with ED, while ED explains unique variance. All encoding performance scores are for the monkey IT electrophysiology data.

References

[1] Michael F Bonner and Russell A Epstein. Computational mechanisms underlying cortical responses to the affordance properties of visual scenes. *PLoS computational biology*, 14(4):e1006111, 2018.

[2] Seyed-Mahdi Khaligh-Razavi and Nikolaus Kriegeskorte. Deep supervised, but not unsupervised, models may explain it cortical representation. *PLOS Computational Biology*, 10(11):1–29, 11 2014. doi: 10.1371/journal.pcbi.1003915. URL `https://doi.org/10.1371/journal.pcbi.1003915`.

[3] David Marr. *Vision: A computational investigation into the human representation and processing of visual information*. MIT press, 2010.

[4] Kim F Nimon and Frederick L Oswald. Understanding the results of multiple linear regression: Beyond standardized regression coefficients. *Organizational Research Methods*, 16(4):650–674, 2013.

[5] Ben Sorscher, Surya Ganguli, and Haim Sompolinsky. Neural representational geometry underlies few-shot concept learning. *Proceedings of the National Academy of Sciences*, 119(43):e2200800119, 2022.

[6] Daniel L. K. Yamins and James J. DiCarlo. Using goal-driven deep learning models to understand sensory cortex. *Nature Neuroscience*, 19(3):356–365, Mar 2016. ISSN 1546-1726. doi: 10.1038/nn.4244. URL `https://doi.org/10.1038/nn.4244`.

[7] Daniel L. K. Yamins, Ha Hong, Charles F. Cadieu, Ethan A. Solomon, Darren Seibert, and James J. DiCarlo. Performance-optimized hierarchical models predict neural responses in higher visual cortex. *Proceedings of the National Academy of Sciences*, 111(23):8619–8624, 2014. ISSN 0027-8424. doi: 10.1073/pnas.1403112111. URL `https://www.pnas.org/content/111/23/8619`.
