# Supplementary material for: High-performing neural network models of visual cortex benefit from high latent dimensionality
Source: PLoS Comput Biol. 2024 Jan 10;20(1):e1011792. doi: 10.1371/journal.pcbi.1011792 (PMC10805290; doi:10.1371/journal.pcbi.1011792)
Supplement: Attachment — Submitted filename: reviewer_responses.pdf [file pcbi.1011792.s011.pdf]

Reviewer responses

We thank all reviewers for their constructive comments, which we have addressed in the revised manuscript. Our point-by-point replies are outlined below in **bold**. Please note that we previously included our main manuscript and appendices in one file, but we have since been asked by the journal to instead upload a supplementary file for each appendix section. We apologize for the confusion caused by this reorganization.

Reviewer 1

First, I'd like to applaud the authors on the beautiful paper. The results and presentation are super clear and I really like it.

Summary

I'm a big fan of using geometric language to explain DNN related phenomena in mechanistic way. The results from this paper gave me further insights on how to interpret the previous neural regression results.

In this paper, the authors clearly pointed out a potentially neglected possibility that some models have better brain predictivity not because their representation is better aligned, but because their effective dimensionality is large.

The authors extensively benchmarked and compared DNN encoding models for neuronal responses in primate IT and human fMRI data from higher visual area. They found that the effective dimensionality of the DNN representation is a robust predictor of its encoding accuracy, across training tasks and datasets, even controlling for layer depth. They also found that the effective dimensionality strongly correlates with the ability of classifying novel categories per few-shot learning.

All in all, this paper provides computational evidence for the benefits of high-dimensional neural code. It also provides a new lens to interpret previous results of brain predictivity.

Strength

- There are extensive controlled experiment for the main effect
1. Results in Fig3B F.1B showed that even when controlled for the training method and the layers, the higher-dimensional representations are better regressors for neurons. This answered my question of "is ED just another name for the depth of the layer", since ED and layer depth are correlated.

2. The results from control experiments in I.1 and I.2 are super interesting, which showed that when trained on label-shuffled ImageNet the DNN acquired lower ED in its representation and lower predictive power of the brain.
3. Further, simply applying a linear transform to whiten the representation increases the Effective Dimensionality (ED) but doesn't enhance the predictive power. This demonstrates that the Effective Dimensionality may not be the ultimate answer; instead, it could be a proxy for something more profound and directly related to the richness of the representation.
- The power law decay of spectra and its relation to the theory in Stringer et al 2019 Nature is surprising and interesting.
- There are extensive citations and links to prior discussions of the dimensionality of the neural code. These references help contextualize the new evidence introduced by this work within the existing literature.

Thank you for this positive assessment! We very much appreciate the reviewer's detailed consideration of the many analyses in this manuscript.

Major Question

However, there is a major issue I had with the interpretation of the data.

Regarding figure F.2 I have some issue with the authors' characterization "*With the exception of the monkey V1 data, we found a strong, positive relationship between ED and encoding performance across multiple datasets*". and they gave their interpretation in L228-234.

This positive correlation is statistically true for V4 and IT, but when looking at the data, there is another interpretation which the authors didn't mention in main text: the relation between ED and R2 looks like an inverse U shape, which is reminiscent of the middle panel of Figure 1.C

If I were the authors, I would not fit a linear line to the data in Figure F.2, instead I'll fit a U shaped curve and inspect the peak of the U shape as the "optimal ED" for this dataset. This would better fit the trend of data for V1 and V4. Using this model, it seems to me that V1 has a smaller optimal ED than V4 and V4 smaller than IT. This will be consistent with two facts: 1) your observation in Fig. H.1 that the log of ED scaled near linearly with the depth of the layer in ResNet. 2) usually the earlier layer in DNN is a better predictor of V1 data and deeper layer better for IT, and the depth of the optimal layer for regressing each cortical area increase along the ventral stream.

Thus the alternative interpretation that can be drawn from the data is that each visual area has an optimal ED, where higher visual cortices are associated with a higher optimal ED. If we focus our attention on the higher visual area where the optimal ED is as high as or higher than what can be obtained from ResNet, we will only observe the rising phase of the inverse U shape. In

other words, the encoding score positively correlates and increases with ED. If this interpretation is correct, it doesn't contradict your main conclusion; rather, it refines your picture and potentially makes it more nuanced and intriguing.

Independent lines of research argue that there is an increase in intrinsic tuning dimension along the ventral stream [1]. This has been characterized using online search approaches to explore neural tuning on a high-dimensional image manifold. The findings indicate that the tuning peaks for the inferotemporal cortex (IT) are sparser, sharper, and have more tuned dimensions than those of V1 (primary visual cortex). This progression in intrinsic tuning dimension, along with the progression of optimal ED of the CNN (Convolutional Neural Network) regressor, seems interconnected and worthy of exploration.

My intuition is that if the neurons are tuned for K feature dimensions and are invariant to the rest, then the CNN's latent dimensions need to have at least K effective feature dimensions to regress it. Consequently, the more tuned dimensions the neurons possess, the higher the dimension the CNN representation needs to have in order to predict it.

[1]: Wang B, Ponce CR. Tuning landscapes of the ventral stream. Cell Rep. 2022 Nov doi: 10.1016/j.celrep.2022.111595

In response, I would like to hear the authors' thoughts on the following points:

- In the revised version of the paper, it would be beneficial if the authors could directly address this alternative view and tailor some conclusions specifically to the higher visual cortex. For instance, the conclusion found in lines 309-311 might need to be revised to focus on the "higher visual cortex" rather than the entire ventral stream.

This is an excellent suggestion and an interesting interpretation of our results. We have now fit inverted-U curves (2nd order polynomial regression) to the data (previously Figure F.2, now Figure F.3), and it is indeed the case that the "optimal" ED increases from V1 to V2 and from V4 to IT (although the curve for IT did not peak to a maximum). Note that we cannot compare these optimal values across the (V1, V2) and (V4, IT) pairs, because the recordings were obtained from different studies that used different stimulus sets, as explained in the caption for Figure F.3.

We have edited the text to directly address the possibility that each region might have an "optimal" ED and that in general this value might increase along the cortical hierarchy. Importantly, in Section 2.3 where we present results on our neural data, we now outline this alternative possibility and the evidence for it coming from Figure F.3. We also now cite the relevant work by Wang and Ponce.

Finally, we have taken your suggestion to revise the description of our main findings throughout the manuscript so that the focus is on the higher level visual cortex rather than the entire ventral stream.

Another approach could involve providing additional validation through neural data.

- With regard to the V1 dataset, I recall the Freeman 2013 paper, which recorded texture responses from both V1 and V2. Is there an opportunity to perform the same evaluation for V2 neurons and incorporate the findings into Figure F.2?

Thank you for the suggestion. We have incorporated results for V2 using recordings from the Freeman 2013 study, and found that it is consistent with our other results. In regards to the previous comment about optimal model ED values for each region, it appears that V2 has a higher optimal model ED than V1, which is consistent with the reviewer's proposal that ED may increase along the visual hierarchy.

- As for the fMRI dataset, could the plot for the V1 area be displayed in the same figure as Figure F.3? Including these additional data from earlier visual cortices will aid in either validating or refuting the question raised above.

We have now included early visual cortex results for our fMRI data in Figure F.4. It turns out that in this case, there is still a strong positive relationship between ED and encoding performance.

Just want to mention that, even if the conclusion is specifically applicable to IT or higher visual cortices, the result and the paper remain highly intriguing. They provide a geometric perspective that allows us to examine prior findings in a new light.

Thank you for this encouraging assessment of our manuscript.

Minor Issue / Question

1. **Neural regression method with a spatial focus**

There are many methods for regressing neuronal responses to CNN activation tensors. As you mentioned, PLS is a good one, but not the only one. You have also showed the OLS result in F.4, which is consistent.

In some sense, the PLS and OLS get rid of the spatial structure of the tensor. Could you possibly provide some pilot data for neural regression using spatial factorized readout? Here are some reference using these kinds of spatial-channel factorized method (It's understood that redoing the whole analysis maybe too costly)

****Neural system identification for large populations separating "what" and "where"****
https://arxiv.org/abs/1711.02653 (Factorized convolution)

****Factorized convolution models for interpreting neuron-guided images synthesis****
https://2022.ccneuro.org/view_paper.php?PaperNum=1034(Factorized correlation / regression)

Generalization in data-driven models of primary visual cortex
https://www.biorxiv.org/content/10.1101/2020.10.05.326256v2 (Gaussian readout)

These methods have been used widely in practise e.g.
https://www.science.org/doi/10.1126/science.aav9436 and
https://www.biorxiv.org/content/10.1101/2023.05.12.540591v1

We agree that this would be a nice analysis to conduct. However, after having reviewed the proposed methods, we found that they would all require us to fit separate functions for each neuron in each layer of each DNN model using SGD. Given that we consider hundreds of layers (each with up to several thousand neurons,) and that we have limited compute resources, we believe that this analysis would be prohibitively expensive to conduct.

2. Neural network architecture diversity.

The paper has conducted extensive experiments utilizing the ResNet architecture, known for its popularity and residual connections. Could it be possible or feasible to incorporate additional CNN architectures into the analysis, such as Inception, VGG, AlexNet, or CorNet? This inclusion might enhance our ability to interpret the Brain Score leaderboard, where a multitude of architectures are evaluated and compared. If we discover that effective dimensionality serves as a robust predictor for neural predictivity within the BrainScore framework, the findings could become even more intriguing and significant.

This is a great suggestion. Originally, we focused on ResNet architectures to control for architectural variation and because we were able to obtain pre-trained ResNet weights for a variety of supervised and self-supervised tasks. However, we agree that it would strengthen the story to show that this effect can also be observed in other popular architectures besides ResNet. We have therefore now included AlexNet, VGG-16, and SqueezeNet models, both untrained and pretrained on ImageNet classification. All relevant figures in our manuscript have been updated to include these additional models. Note, however, that whenever we show layer-controlled results (e.g., Fig. 3b) we do not include these additional architectures because there is not a one-to-one correspondence of layers across different architectures. For the reviewers' reference, we have included a color-coded

version of Fig. 3a below with the newly added CNN models indicated in orange.

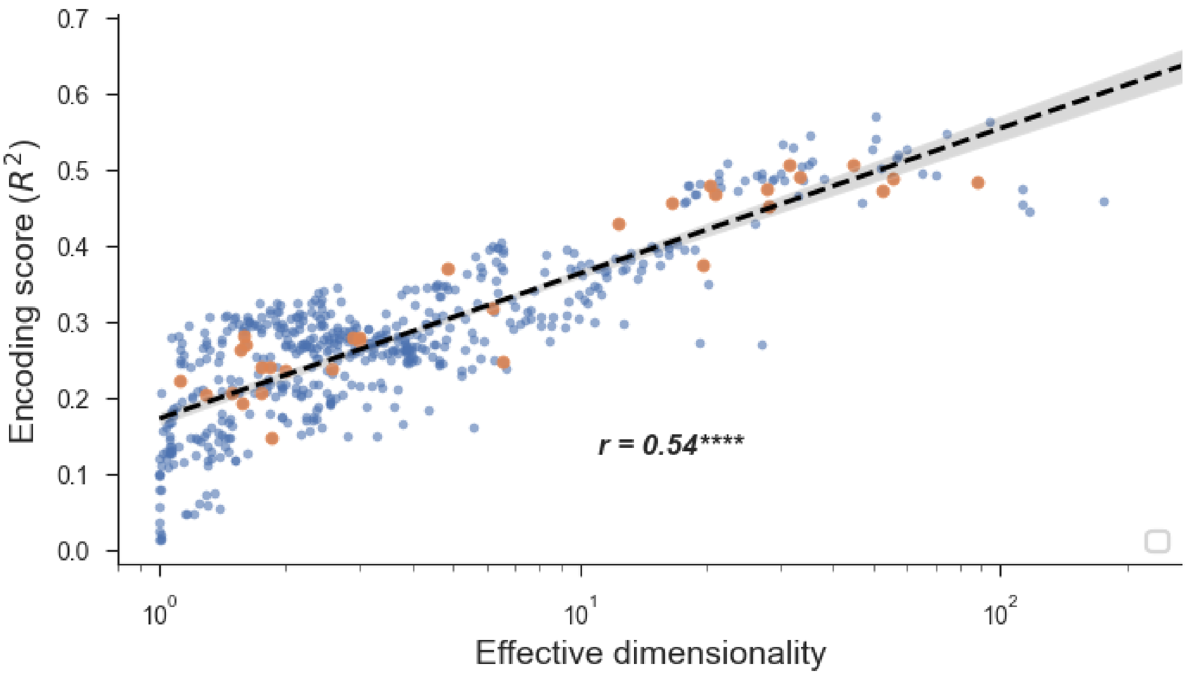

Version of Figure 3a showing newly added models in orange. This plot shows the relationship between effective dimensionality and encoding performance for the monkey IT brain region.

3. **Method details regarding projection distance.**

In L480-485, the authors described how they computed the normalized projected deviation. I'm still unsure if I can reproduce it by reading this text. specifically, I'm not sure what "mean standard deviation along all category subspace dimensions" refers to.

Maybe a few equations specifying how the normalization is done will be helpful.

Further I saw no method section corresponding to Figure 6b, a simple description of the experimental set up will be helpful.

In the Methods sections, we have added details describing how the normalized projection distances and the simulated data in Figure 6b were computed, including all relevant equations.

4. In the main text, there's a statement concerning projection that I find unclear (lines 285-288). Specifically, the parenthetical comment "*(assuming that the radius is held constant)*" leaves

me uncertain about what the term "radius" refers to in this context. In my understanding, the quantity that should be held constant is the summed or averaged variance across all dimensions. By distributing this variance over more dimensions, the variance on an individual projection dimension would be significantly reduced. Is my interpretation consistent with what the authors intended?

This interpretation is exactly what we intended for the empirical data in our models, and we have clarified the methods section to make this explicit with relevant equations. However, for the particular parenthetical passage above, we were talking about the radii of idealized spherical class subspaces (which is what we used to generate the simulated toy example in Figure 6b). We have clarified the text by removing the parentheses and saying "If we consider an idealized scenario in which category instances are distributed uniformly within *unit* spheres", so that the sphere radius is understood to be the value which is held constant.

5. Regarding the organization of the paper, it might be beneficial to add a separate subsection dedicated to discussing Effective Dimensionality in the context of Deep Neural Network (DNN) representation. For example, a focus on the factors that influence ED would be quite interesting, and it would assist readers in evaluating the relationship between higher ED and increased predictivity. It would be valuable to explore in this section why using ED as a proxy for representation richness is effective, as well as what might undermine this approach. Lines 184-206 and 207-211 have already provided a concise version of this idea, but further elaboration could enrich the understanding of the topic.

This is a very helpful suggestion. We have taken up this suggestion and added a new section to the Results called "Dimensionality in deep neural networks" (Section 2.2 in the revised manuscript). We believe that the expanded treatment of these topics is a major improvement to the manuscript.

6. On line 475, the reference to "Appendix 3" appears to be a typo or mislink, as there is no such appendix in the document. Please consider correcting this reference.

We have corrected the reference. Thank you for spotting this.

7. There seems to be a typo on line 468 involving "N_{test}". Please review and correct as needed.

We have corrected the typo.

Reviewer 2

Elmoznino and Bonner's study is a valuable contribution to the field, shedding light on the relationship between the geometry of Deep Neural Networks (DNNs) and their performance. They challenge existing notions by demonstrating that DNNs with higher-dimensional representations offer better explanations for cortical responses and enhanced adaptability to new training domains. The authors substantiate their claims using an impressive blend of multi-unit recordings from monkeys and BOLD-MRI data from humans, coupled with an extensive collection of resnets trained on both Imagenet and Indoor buildings datasets. The findings consistently reveal that increased effective dimensionality correlates with improved neural data explanation, particularly in monkey IT, though with varied strength across other areas such as V1, V4 and human BOLD-MRI.

The paper excels in introducing alignment pressure, effective dimensionality, and their potential relevance to encoding performance and feature learning. This makes it accessible even to readers less familiar with these concepts. Nevertheless, I have some substantial concerns and some minor points that warrant attention:

Thank you. We are glad that the reviewer considers this to be a valuable contribution to the field, and we appreciate the reviewer's positive comments on the extensive analyses and results presented in this paper.

Major Concerns

1) The paper predominantly highlights the pronounced pattern in monkey IT, while comparatively weaker effects are observed in V4 and human BOLD-MRI. Not showing at least some of this data in the main text is somewhat misleading.
2) The assertion that "network layers with a slower decay in eigenspectrum are better in explaining neural data" primarily holds for monkey IT, while weaker associations are seen in V1, V4, and human BOLD-MRI. Only presenting IT in the main text is misleading.

We have now included the human BOLD-MRI results for the lateral occipital region (LO) in the main text. We believe that including additional results for more regions in the main text beyond this would make the presentation too cluttered. However, we have edited the entire manuscript so that our statements specifically focus on high-level visual cortex, rather than visual cortex in general. We have also added a new paragraph in the Results section that describes our findings in other brain regions. This new paragraph explicitly states that the findings were strongest in high-level visual cortex and that weaker effects were observed in other regions, with the exception of V1, where there was no effect. This new paragraph also includes a discussion of the possible interpretation that the optimal ED increases across the visual hierarchy (a possible interpretation that was mentioned by both

Reviewers 1 and 2). We believe that these changes clarify that our claims are specific to high-level visual cortex, and we hope that these changes address the reviewer's main concern about how the results are presented.

3) The authors propose that higher-tier areas, processing input from lower-tier areas, benefit most from higher dimensionality due to the robustly low-dimensional nature of the latter. This suggests a potential pattern wherein lower-tier areas exhibit lower dimensionality than higher-tier ones. Addressing this possibility and its potential implications vis-à-vis previous research would add depth to the discussion.

This is a great suggestion, and, in fact, a related idea was raised by Reviewer 1. To address the possibility of a relationship to ED that depends on depth in the cortical hierarchy, we have now fit inverted-U curves (2nd order polynomial regression) to the data (previously Figure F.2, now Figure F.3), and found that the "optimal" ED increases from V1 to V2 and from V4 to IT (although the curve for IT did not peak to a maximum). Note that we cannot compare these optimal values across the (V1, V2) and (V4, IT) pairs, because the recordings were obtained from different studies that used different stimulus sets, as explained in the caption for Figure F.3.

We have edited the Results section to directly address the possibility that each region might have an "optimal" ED, and that in general this value might increase along the cortical hierarchy. Importantly, in Section 2.3 where we present results on our neural data, we now outline this possibility and the evidence for it coming from Figure F.3.

4) I miss a discussion of how results that show that the brain is low-dimensional could have been observed.

Our results cannot speak to the dimensionality of neural representations in the brain but rather representations in computational models of the brain. Indeed, even if the brain were low-dimensional, the theory that we developed in Section 2.1 of our paper predicts that high-dimensional DNNs should nevertheless better explain neural activity, in expectation. Our original submission briefly addressed this point in a paragraph of the Discussion section. In our revised manuscript, we have now edited this paragraph to make this important point more explicit. Below we quoted the relevant section of the Discussion:

"Our results raise several important considerations for future work. First, while our findings show that computational models of visual cortex benefit from high latent dimensionality, our method cannot speak to the dimensionality of visual cortex itself and was not designed to do so. Indeed, the theory that we presented in Section 2.1 predicts that high-dimensional DNNs should generally better explain neural activity *even if neural representations in the brain are low-dimensional*. "

5) No V1 BOLD-MRI data is shown. This would be informative (maybe use an anatomical mask to get it if a field map is absent).

We have now included early visual cortex results for our fMRI data in Figure F.3. It turns out that in the fMRI data, there is still a strong positive relationship between ED and encoding performance.

6) The authors use resnets because the convets are better comparable. That is reasonable but resnets have a very specific architecture. Would it be sensible to look at some other types of models with this as caveat? At least it would be good to observe that the pattern, higher ED results in higher encoding score is also present in a not skip-connection architecture.

This is a great suggestion. Below is a copy of our response to a similar comment from Reviewer 1:

Originally, we focused on ResNet architectures to control for architectural variation and because we were able to obtain pre-trained ResNet weights for a variety of supervised and self-supervised tasks. However, we agree that it would strengthen the story to show that this effect can also be observed in other popular architectures besides ResNet. We have therefore now included AlexNet, VGG-16, and SqueezeNet models, both untrained and pretrained on ImageNet classification. All relevant figures in our manuscript have been updated to include these additional models. Note, however, that whenever we show layer-controlled results (e.g., Fig. 3b) we do not include these additional architectures because there is not a one-to-one correspondence of layers across different architectures. For the reviewers' reference, we have included a color-coded version of Fig. 3a below with the newly added CNN models indicated in orange.

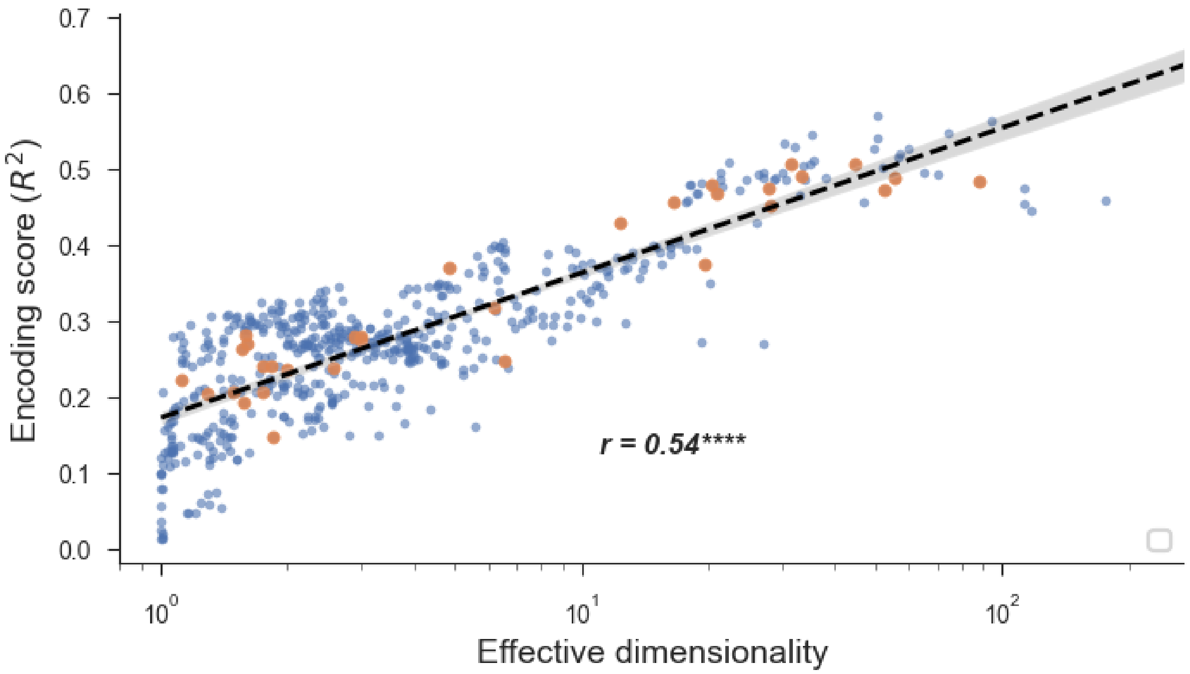

Version of Figure 3a showing newly added models in orange. This plot shows the relationship between effective dimensionality and encoding performance for the monkey IT brain region.

Minor Remarks

Page 3, Line 61: Reframing "computational models of visual cortex" to "models that explain neural data" offers greater precision in describing the authors' contributions.

We have changed the phrasing to: "we found that DNNs with higher latent dimensionality explain more variance in neural data".

Page 6: The caption of Figure 1 refers to a "Joint regime" containing two plots, yet these are not separately labeled within the caption.

We have now improved the clarity of the figure caption by separately identifying the two "Joint regime" plots as one that corresponds to "low dimensionality" and another that corresponds to "intermediate dimensionality", respectively.

Page 9, Line 215: Emphasize that although the analysis in 3b partly addresses differences in effective dimensionality across DNN layers, some distinctions may still arise due to inherent variations between layers.

We have edited the text to address this point (as we understood it), but please let us know if we misunderstood the caveat you had in mind. The edited text now reads:

“This effect was also observed when separately examining subsets of models from the same DNN layer (Fig. 3b), which means that the relationship between ED and encoding performance cannot be reduced to an effect of depth. (Note that the reverse is also likely true: there may be effects of depth that cannot be reduced to effects of ED.)”

Page 10: For Figure 3c, enhancing the color differentiation between the three conditions would aid clarity.

Thank you for the suggestion, we have changed the figure to use more differentiated colors.

Page 11: Given the exceptional performance of the area illustrated in Figure 4, it's advisable to mitigate potential misinterpretation by providing context regarding its superiority.

We have now clarified in the caption for Figure 4 (and Figure 3) that we are showing encoding performance for the IT brain region, and we also say that this was the region with the strongest trend between ED and encoding performance.

Page 14, Line 317: Consider specifying that the downstream readout could pertain to V1.

We have incorporated this suggestion in the Discussion section.

Page 30: In Appendix C, abbreviations of formulas spanning four pages, with only "AP" defined in the main text, might benefit from being introduced closer to Figure C1 for improved coherence.

We have moved Figure C1 lower down in the text to be closer to the variable definitions that appear in the figure.

In conclusion, Elmoznino and Bonner's paper is a captivating exploration of DNN geometry's connection to performance, encapsulating both significant insights and areas necessitating further clarification.

Thank you for this positive and encouraging assessment of our manuscript!

Reviewer 3

This paper by Elmoznino and Bonner aims to explain the success of deep neural networks (DNNs) as computational models of vision. The standard explanation to this end has been “task performance” which is typically some kind of engineering benchmark like top X% accuracy on Imagenet. Here the authors suggest an alternative approach – dimensionality, which is basically

the sparseness of the eigen-spectrum. This understanding could potentially lead to the development of better models of vision which would be impactful. I commend the authors on making their code etc open access which makes it much easier to review the study and is a contribution to the field. This is a high standard that others should aspire to. But there also are several issues, including a lack of replication of the reported effects (Conwell 2023), that dampen the impact of the authors' proposal (see below).

The core claim of this study is that neural networks with high dimensionality are better computational models of vision. My review will be focused almost entirely the authors' defense of this central claim.

Major issues

1. Issues with the dimensionality metric for DNNs. There are at least 3 issues around the use of this specific metric (effective dimensionality, ED).

a. ED estimates the sparseness of the eigen spectrum of the covariance matrix of the data. Essentially this is an analytical way to remove the low magnitude eigen values (noise) from the high magnitude ones (signal). (Relatedy this is a very old metric that pre-dates citation 32. Please cite correctly). This metric makes sense when estimating dimensionality from noisy sources, like neural or fMRI data. It is unclear why the authors would choose to apply the same metric for a noiseless data source like a neural network.

We have added the earlier references on ED. Regarding the applicability of ED in deterministic models such as DNNs, we agree that the theoretical motivation is more nuanced. In the Results section of our original submission, we briefly addressed the theoretical and empirical evidence for why ED can be a useful metric for characterizing DNN representations. However, based on this reviewer comment and a related comment from Reviewer 1, we have now added a dedicated section to the Results in our revised manuscript that more thoroughly addresses the motivation for examining ED in DNNs and includes additional relevant citations (Section 2.2 "Dimensionality in deep neural networks"). Below, we have copied the relevant portion of the text that directly addresses this comment:

"Before presenting our analyses, it is helpful to first consider the interpretation of latent dimensionality in the context of DNNs and to highlight some important methodological details. The logic underlying dimensionality metrics like ED is that the scale of a dimension's variance is indicative of its meaningfulness, with high variance corresponding to meaningful dimensions and low variance corresponding to random or less-useful dimensions. Even in deterministic systems, like the DNNs examined here, variance scale can indicate meaningfulness. Indeed, previous work has shown that network training

expands variance along dimensions that are useful for solving tasks while leaving unaltered, or even contracting, variance along random dimensions [29, 72, 26, 41, 89]. This explains why networks trained with different random initializations end up with similar high-variance principal components but different low-variance components, and it explains why measures of network similarity appear to be more meaningful when they are weighted by variance [51, 56]. Furthermore, these empirical observations are consistent with theories of deep learning which argue that networks progressively learn the principal components of their training data and tasks, with the number of learned components increasing as a function of task and data complexity [41, 42, 36, 7, 76, 56]. "

Note that we also provide additional discussion of the theory of dimensionality in DNNs in Appendices A & I (now Supplementary files S1 & S9).

b. The second concern is the specific way in which the ED metric was applied to DNN activations. Here the authors first apply a global pool to the convolutional maps from the DNNs before computing ED. This specific experimenter choice can completely change the overall inference about the dimensionality in the study. As an example, the authors discuss the untangling hypothesis across successive stages of the hierarchy (Cox, DiCarlo and others). They further and use the results of this study to argue that dimensionality in fact increases or gets more entangled (and not decreases, or less untangled) along the hierarchy. This comparison is not warranted because those previous studies were motivated by increase in invariance (mostly size, position and viewpoint which are encoded in spatial parameters) which the pooled version would not capture.

We agree that this is an important methodological difference between our work and some of the previous work on dimensionality in vision. For this reason, we have added a new paragraph to the Discussion that addresses this difference. This paragraph is copied below:

"We should also note that because we focused specifically on channel covariance in our main analyses, our notion of dimensionality is different from the dimensionality metrics used in most previous studies of CNNs, which generally did not make a distinction between channel and spatial information [e.g., 3, 19]. We believe that channel and spatial covariance have different functional consequences for visual representations, and it may be important to keep the distinction between these two factors in mind when examining dimensionality and when comparing results across studies. Nonetheless, we did not find evidence in our study to suggest that dimensionality trends in CNNs are markedly different when spatial information is included—-rather when including spatial information in our analyses, we found qualitatively similar, though weaker, relationships between dimensionality and measures of model performance. We suggest that a useful direction for future work is to better understand the specific ways in which channel and spatial covariance might differentially affect task performance in neural networks."

We would also like to mention several key points here in regard to this comment:

- **While it is true that we observed an increase in ED at higher layers, this finding was only shown in the Appendix, and it was not used to support the main theoretical arguments in our manuscript. Our central claim is not about the way in which ED changes along the computational hierarchy. Rather, our claim is that models with higher dimensionality can better explain neural activity in high-level visual cortex.**
- **We also conducted analyses without spatial pooling and found qualitatively similar results, which were reported in Appendix F (now Supplementary file S6) and mentioned in Section 2.2. Thus, while we believe that pooled activations provide a better lens on the richness of neural network representations, our central findings are, nonetheless, similar even without global pooling.**
- **Previous work has indeed argued that dimensionality reduction along the hierarchy supports viewpoint-invariant recognition. However, it is not true that pooled layers are unable to capture aspects of viewpoint invariance. CNN layers are invariant to translations by design once pooling is applied, but not to other aspects of viewpoint, such as size and pose—just translation. Translational invariance, in turn, is only a small component of viewpoint-invariant recognition.**
- **We also want to note that dimensionality and entanglement are not the same. It is possible to have a representation that is both high-dimensional and disentangled, so long as the dimensions encoding task-relevant stimulus features are linear and orthogonal to task-irrelevant stimulus features.**

c. The third related concern about the choice of global pooling before estimating ED is that restricts the analysis to convolutional models only. As such this specific test would not be possible in older model classes (like hmax) or newer, SOTA models like transformer. This significantly limits the scope of this method to conv nets alone.

This is a good point. We have now added the following text to the Discussion to specifically address this limitation:

“Note that our approach of using global pooling to focus on channel covariance means that our methods are tailored to convolutional architectures. Thus, further work will be needed to apply our approach to other classes of architectures in which channel and spatial information are not readily separable.”

We would like to add, however, that we believe that tailored statistical estimators (such as our channel dimensionality metric) and controlled computational studies are important for revealing phenomena that may otherwise be difficult or impossible to observe when extraneous factors of variation are left uncontrolled. Indeed, it is a standard paradigm in computational neuroscience to investigate neural computation through studies carried out

under simplified and controlled settings. We worry that without such controlled studies, neuroscientists run the risk of constantly chasing after SOTA models coming out of the engineering field rather than seeking a deeper understanding of the underlying principles of neural representation.

2. Issues with the central claim of the study. The authors here consider every layer of every base model (architecture + training regime) to obtain the key figure supporting the central claim of the study (Figure 3). As such every layer is considered as a "model" of the visual system. This is a very specific interpretation of a model of visual cortex and is otherwise highly unusual because most others would consider the best layer for a given base model as a putative model.

a. Similar correlation plots for best-layer only across the model set (for untrained and trained models separately)

b. Similar correlation plots for the best-layer only, without the global pooling

Thank you for the suggestion. We have now included the results asked for in comments (a) and (b) in Supplementary file S6. The plots show the ED and encoding performance for only the best-performing layer in each base model, as is done in some of the existing literature. Both with and without pooling, our main results continue to hold (although the effective sample size for this analysis is more limited, n=46 models). Note that we opted not to show separate analyses for untrained models, as the sample size would have just been n=5 models, which we do not believe has much value. As a result, the plots we have shown include both the 41 trained and the 5 untrained models.

c. Generalization beyond Taxonomy models. Taxonomy models are in general bad models of the high-level vision (see Conwell 2023 arxiv, Murty 2022 Nature Comm)

ImageNet-trained models are included in our analyses, but we had not previously examined the relationship between ED and encoding performance separated by training set. In our revision, we have now included these results in Supplementary file S6, which show that the effect is also observed looking at only ImageNet-trained models.

d. A discussion on how this study relates with the recent Conwell (2023 arxiv) paper that did not find any relation between model encoding performance and latent dimensionality (Figure 5 and associated sections). It is important to reconcile a direct failure to replicate the stated claims of this report.

We have now added a paragraph to the Discussion that addresses the differences between our work and the Conwell et al. study. The most important point is that the Conwell study does not control for architecture and that ED values do not have the same meaning across

highly different architectures. Below is a copy of this paragraph, which addresses these issues in detail:

"A recent preprint examined the ED of layer activations across a highly varied set of neural networks and found that while ED was higher for trained versus untrained versions of the same architectures, as was also shown here, variation in ED across diverse trained networks was not correlated with encoding performance in visual cortex [20]. However, we want to emphasize that this latter finding is not unexpected given the nature of the model set examined in the study. Specifically, the authors examined the best performing layer from models with diverse architectures and varied normalization layers, including convolutional networks, transformers, and multilayer perceptrons, all of which can have architecture-specific effects on the shape of representational eigenspectra (see Results 2.2 and Appendix I [note: now Supplementary file S9]). Importantly, the existence of such architecture-specific effects means that a given ED value will not have the same meaning across all networks, and, for this reason, we caution against comparing ED values (and related dimensionality metrics) across networks with highly varied architectures [35]. Furthermore, the ED metric computed in Conwell at al. is different from the ED metric that we found to be most informative in our own analyses. Specifically, we computed ED based on channel covariance by using global pooling, whereas Conwell at al. computed ED based on a mixture of both channel and spatial covariance, which in our models predicts encoding performance less well (Appendix F [note: now Supplementary file S6]). We focused on channel covariance because it reflects the richness of feature types in a network and because it has been shown to be highly informative for theories of learning in convolutional architectures [41]. In contrast, spatial covariance reflects the spatial smoothness of layer activations, which is informative in its own right but appears to be less useful for understanding the richness of DNN representations. In sum, we argue that the most informative approach for understanding the role of dimensionality in DNNs is to examine how variations in dimensionality are related to variations in model performance while controlling for architecture. We further propose that channel covariance, rather than spatial covariance, offers the best lens on the key representational properties of DNNs."

Note that we have also added a detailed explanation of these important methodological points in Section 2.2 ("Dimensionality in deep neural networks"). Below is a copy of the relevant text:

"It is important to note, however, that the scale of activation variance is not universally indicative of representation quality in DNNs. First, architecture-specific factors can affect ED in ways that are independent of learning, which means that ED values (and related dimensionality metrics) should only be compared across models with similar architectures [35]. Second, it is possible to arbitrarily rescale the variance of any dimension through normalization procedures (see S9). Thus, to gain a better understanding of whether

high-performing DNNs have expressive, high-dimensional representations or compressed, low-dimensional representations, it is important to examine the role of dimensionality while controlling for architecture. Here we controlled for architectural factors by focusing on a set of standard convolutional architectures (mostly ResNet). We provide a more detailed discussion of these points in S1 & S9."

e. A comparison of effective dimensionality as an explanation of better model performance versus other competing hypotheses (for eg. engineering benchmarks like those on brain-score). The authors lay out the other explanations in the discussion (lines 338 onward) but do little to actual test these competing ideas directly.

Thank you for this suggestion. First, we want to clarify an important point: we do not view geometric properties (such as ED) and task performance metrics as representing competing theories but, rather, different levels of explanation. In our revision, we have addressed this point through new analyses that provide additional insights into the relationship between ED and measures of model performance (including performance on both object classification and neural encoding) and through additional theoretical discussion. Specifically, we have added the following in Supplementary file S10:

1. **Analyses of the relationship between ED, classification performance, and neural encoding performance, including variance partitioning analyses.**
2. **Analyses comparing ED to other geometric properties of representations that have been proposed in previous work from Sorscher et al., 2022.**
3. **A detailed explanation of our theoretical perspective on the relationship between geometric properties and measures of model performance.**

Below is a summary of the key take-aways, which are addressed in more detail in Supplementary file S10:

First, as expected, object classification performance correlates strongly with encoding performance. However, it is important to note that task performance and ED are not competing theories but, rather, different levels of explanation that provide complementary insights into the nature of neural network representations. Models that perform ethologically-relevant visual tasks are likely to explain neural responses, because the brain is optimized for such tasks as well and the space of good solutions is likely limited. However, this does not tell us *how* the models solve these tasks, for which we must look at the underlying representational structure. In the language of Marr, theories based on classification performance function at the abstract computational level, but there must also be explanations for DNN encoding performance that function at the representational level. ED is one such representation-based explanation. In Sections 2.4 and 2.5, we showed how ED empirically predicts classification performance on novel categories and provided one

theoretical explanation for why this happens, which was proposed by Sorscher et al., [85]. Furthermore, when we conducted a variance partitioning analysis of encoding performance as a function of both ED and classification performance (Fig. J.1b), we found that nearly all of their explained variance was shared. In sum, there is good reason to believe that ED and classification performance (or visual task performance more generally) are causally intertwined, and that they jointly provide a richer explanation of DNN models of visual cortex.

Second, when comparing ED to other geometric properties of model representations, we found that ED-related properties had the strongest relationship to encoding performance, with the exception of a metric called SNR that aggregates many of the other metrics (and incorporates ED). We also conducted a variance partitioning analysis of encoding performance using ED and SNR as predictors, and found that almost all of their explained variance was shared. Therefore, among several hypotheses based on representational geometry that we tested, ED had the most explanatory power and almost completely accounted for the performance of SNR.

We thank the reviewer for this helpful comment, which we believe has significantly improved the clarity and rigor of our paper.

Overclaims. The paper tends to overclaim the results in several places which must be addressed. For instance, the high positive correlation as such is only observed for high-level visual cortical regions. Low level regions (like V1) often show no relation with effective dimensionality. One can observe the pareto-ceilings even for V4 which has much lower correlation (probably drives by untrained models?. My strong advice is to tone down the claims of the study.

We have now toned down our claims throughout the paper (Introduction, Results, and Discussion sections). In particular, when initially presenting our main findings, we explicitly state that the effects are observed most strongly in higher-level regions and that there is no correlation in V1. Thereafter, we qualify our claims by specifying that we observe a relationship for *high-level* visual cortex.
